# A Serotype-Specific and Multiplex PCR Method for Whole-Genome Sequencing of Dengue Virus Directly from Clinical Samples

**DOI:** 10.1128/spectrum.01210-22

**Published:** 2022-09-12

**Authors:** Wenzhe Su, Liyun Jiang, Weizhi Lu, Huaping Xie, Yimin Cao, Biao Di, Yan Li, Kai Nie, Huanyu Wang, Zhoubin Zhang, Songtao Xu

**Affiliations:** a Guangzhou Center for Disease Control and Prevention, Guangzhou, China; b Institute of Public Health, Guangzhou Medical University & Guangzhou Center for Disease Control and Prevention, Guangzhou, China; c Shandong Center for Disease Control and Prevention, Jinan, China; d Department of Arboviruses, NHC Key Laboratory of Biosafety, National Institute for Viral Disease Control and Prevention, Beijing, China; e State Key Laboratory for Infectious Disease Control and Prevention, Beijing, China; Emory University School of Medicine

**Keywords:** dengue, dengue virus, whole-genome sequencing, next-generation sequencing, multiplex PCR

## Abstract

Dengue virus (DENV) is the most globally prevalent member of the genus *Flavivirus* in the family *Flaviviridae*, which can be classified into four serotypes. Historically, molecular epidemiological studies of DENV depended on E gene sequencing. The development of next-generation sequencing (NGS) allowed its application to viral whole-genome sequencing (WGS). In this study, we report the improvement of the existing WGS process for DENV by optimizing the primer design procedure, designing serotype-specific primer panels and reducing the sizes of amplicons. A total of 31 DENV-positive serum samples belonging to 4 serotypes and 9 genotypes of DENV were involved in the validation of the primer panels. The threshold cycle (*C_T_*) values of these samples ranged from 23.91 to 35.11. The validation results showed that the length of consensus sequences generated at a coverage depth of 20× or more ranged from 10,370 to 10,672 bp, with 100.00% coverage of the open reading frames and 97.34% to 99.52% coverage of the DENV genome. The amplification efficiency varied across amplicons, genotypes, and serotypes of DENVs. These results indicate that the serotype-specific primer panels allow users to obtain the whole genome of DENV directly from clinical samples, providing a universal, rapid, and effective tool for the integration of genomics with dengue surveillance.

**IMPORTANCE** Dengue virus (DENV) is becoming the most globally prevalent arbovirus. The number of people living under the threat of DENV is increasing year by year. With the development of next-generation sequencing (NGS) technology, whole-genome sequencing (WGS) has been more and more widely used in infectious disease surveillance and molecular epidemiological studies. DENV population sequencing by NGS can increase our understanding of the changing epidemiology and evolution of the DENV genome at the molecular level, which demands universal primer panels and combination with NGS platforms. Multiplex PCR with a short-amplicon approach proved superior for amplifying viral genomes from clinical samples, particularly when the viral RNA was present at low concentrations. Additionally, DENV are known for their genetic diversity within serotype groups and geographical regions, so the primer panels we designed focused on universality, which would be useful in future local DENV outbreaks.

## INTRODUCTION

Dengue virus (DENV) is the most globally prevalent member of the genus *Flavivirus* in the family *Flaviviridae*, which can be classified into four serotypes (DENV-1 to DENV-4) according to their serological characteristics (1, 2). Each serotype can be furtherly classified into several genotypes based on the diversity of envelope (E) gene sequences (3–10). The DENV genome is a single-stranded positive-sense RNA of about 10.7 kb. One single open reading frame (ORF) encodes three structural proteins (C, M/prM, and E) and seven nonstructural (NS) proteins (NS1, NS2A, NS2B, NS3, NS4A, NS4B, and NS5) (1, 4, 11). Before being discovered by humans, DENV had been circulating among sylvatic nonhuman primates in the tropics and subtropics for years. First discovered in the 1940s, DENVs have been transmitted gradually to previously unexposed regions over the past 5 decades (12). As a mosquito-borne virus, DENV has two main vectors, namely, Aedes albopictus and Aedes aegypti (13, 14). With the slow rise in global temperatures and the spread of *Aedes* spp., introductions and consolidations of DENVs have been reported in over 100 countries and regions (1, 2, 15–17).

The clinical manifestations of DENV infection range from asymptomatic infection and mild dengue fever (DF) to severe DF, such as dengue hemorrhagic fever (DHF) and dengue shock syndrome (DSS) (18). Primary infection with one serotype of DENV usually results in asymptomatic infection or mild DF with serotype-specific, lifelong, and protective immunity. However, due to antibody-dependent enhancement (ADE), secondary or heterologous infection of DENV may lead to a severe DF (19). The annual number of global DENV infection cases was estimated to be 390 million in 2010, and the urban circulation of one or more DENV serotypes has been observed in some tropical cities (12, 20). DENV infection is becoming an increasingly important infectious disease of global public health significance (2, 15).

In recent years, high-throughput sequencing technology, also known as next-generation sequencing (NGS), has been more and more widely used in infectious disease surveillance (21). There are two different strategies in the use of NGS for molecular diagnosis of infectious diseases: metagenomic sequencing and targeted amplicon sequencing. Theoretically, metagenomic sequencing can produce the whole-genome sequence (WGS) of an emerging, unknown pathogen with an immense depth of sequencing coverage and massive data output, along with the generation of substantial host or background genome data and a more expensive price tag (22, 23). For a known pathogen, targeted-amplicon sequencing is more efficient, with higher accuracy (24). Based on NGS platforms, whole-genome sequencing methods for some viruses have been developed and used in sequencing-based surveillance of both endemic and emerging diseases, such as seasonal influenza, Hantaan orthohantavirus, severe acute respiratory syndrome coronavirus 2 (SARS-CoV-2), Ebola, and Zika virus (22, 24–29). However, the amplification efficiency, sequencing coverage, depth, and repeatability at low RNA concentrations and/or with degraded RNA templates still need to be improved.

Hereby, an online tool named Primal Scheme was developed for designing multiple PCR primer panels with multiple reference sequences, which provides a convenient tool for targeted-amplicon sequencing (24). The amplicon size can be set manually according to the supporting sequencing methods. However, Primal Scheme requires a maximum sequence divergence of not more than 5%, which forces users to separate the primer panels into multiple independent schemes when the references are more divergent, such as those for DENV, chikungunya virus (CHIKV), and norovirus.

Our method aims to support the DENV genomic surveillance network and improve the existing WGS process for DENV by optimizing the primer design procedure, designing serotype-specific primer panels, and reducing the size of amplicons.

## RESULTS

### Primer design.

**FIG 1:**
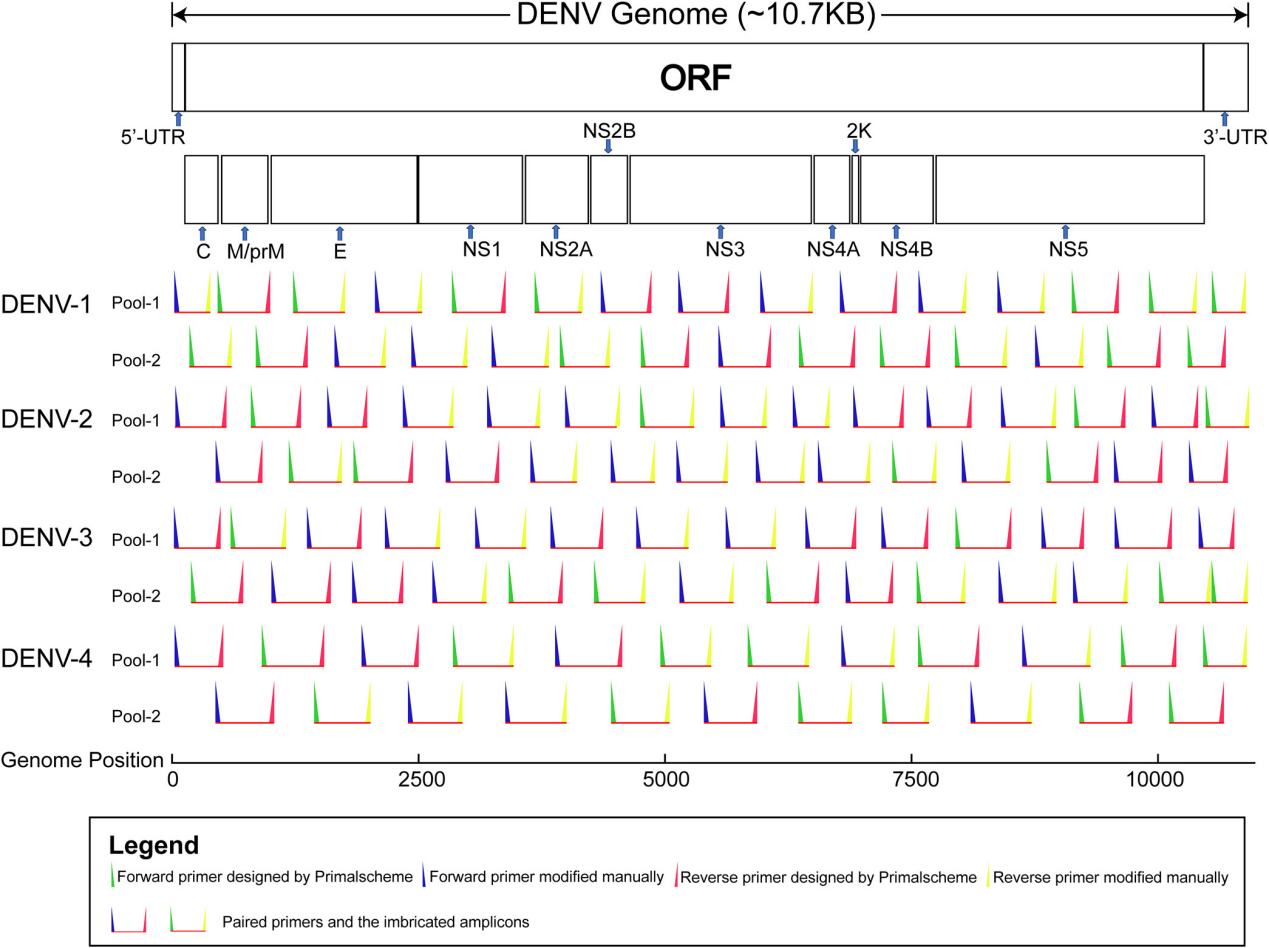
Imbricated design of the multiplex PCR primer panels for DENV-1 through DENV-4. Odd-numbered and even-numbered primers were divided into primer pools 1 and 2, respectively. The genome positions of DENV-1 through DENV-4 were based on NCBI reference sequences (GenBank accession numbers NC_001477, NC_001474, NC_001475, and NC_002640, respectively). Primers in the same rows belong to the same pool. The length of the horizontal red lines represents the approximate size of the imbricated amplicons. The intended overlap regions between pools are also presented. UTR, untranslated region.

**FIG 2:**
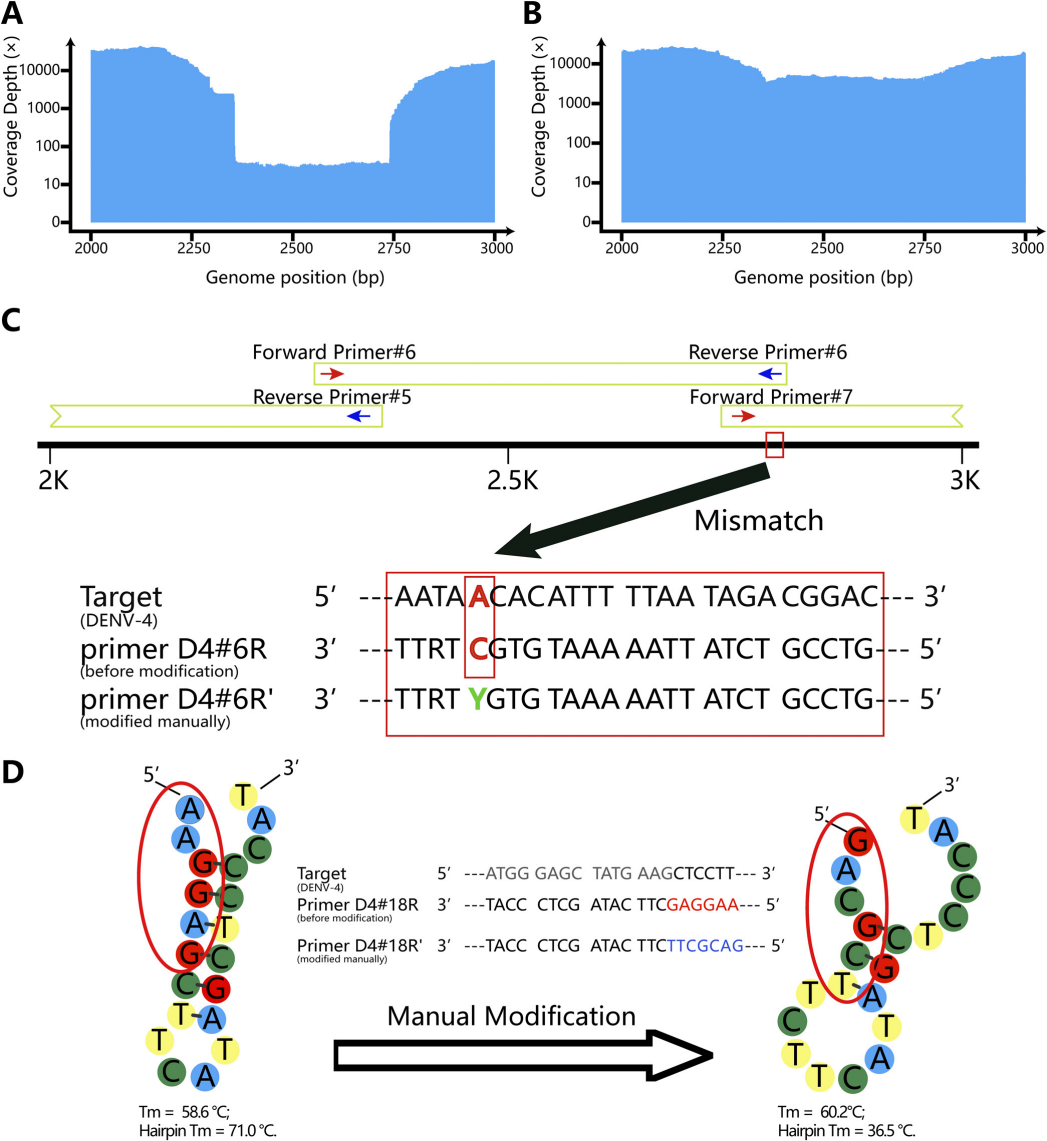
Strategy for improvement by manual modification of primers. (A) Coverage depth of a DENV-4 isolate (GenBank accession number MW881530) at position 2300 to 2750 before manual modification of primer D4#6R; (B) coverage depth of a DENV-4 isolate (MW881530) at position 2300 to 2750 after manual modification of primer D4#6R; (C) manual modification of primer D4#6R at the fifth site by replacing the “C” with a “Y”; (D) manual modification of primer D4#18R on the 5′ end lead to the change of hairpin *T_m_*.

After several rounds of computational simulation and pre-experiment, the primer panels for each DENV serotype were finally set up (Fig. 1; see also Table S1 in the supplemental material). For multiple PCR of DENV-1 through DENV-4, we used 58, 58, 56, and 46 primers, respectively. The design lengths of the primer panels for DENV-1, 2, 3, and 4 were 10,717 bp, 10,715 bp, 10,682 bp, and 10,626 bp (reference sequence coverage, 99.83%, 99.93%, 99.77%, and 99.78%), respectively. To maximize the universality of the primer panels, we used primers with degenerate bases when necessary. Additionally, manual modification near the 5′ end of primers can effectively prevent primer self-dimers and hairpin structures with a high melting temperature (*T_m_*). The manual modification strategies and improvements are shown in Fig. 2.

### Genome coverage.

**FIG 3:**
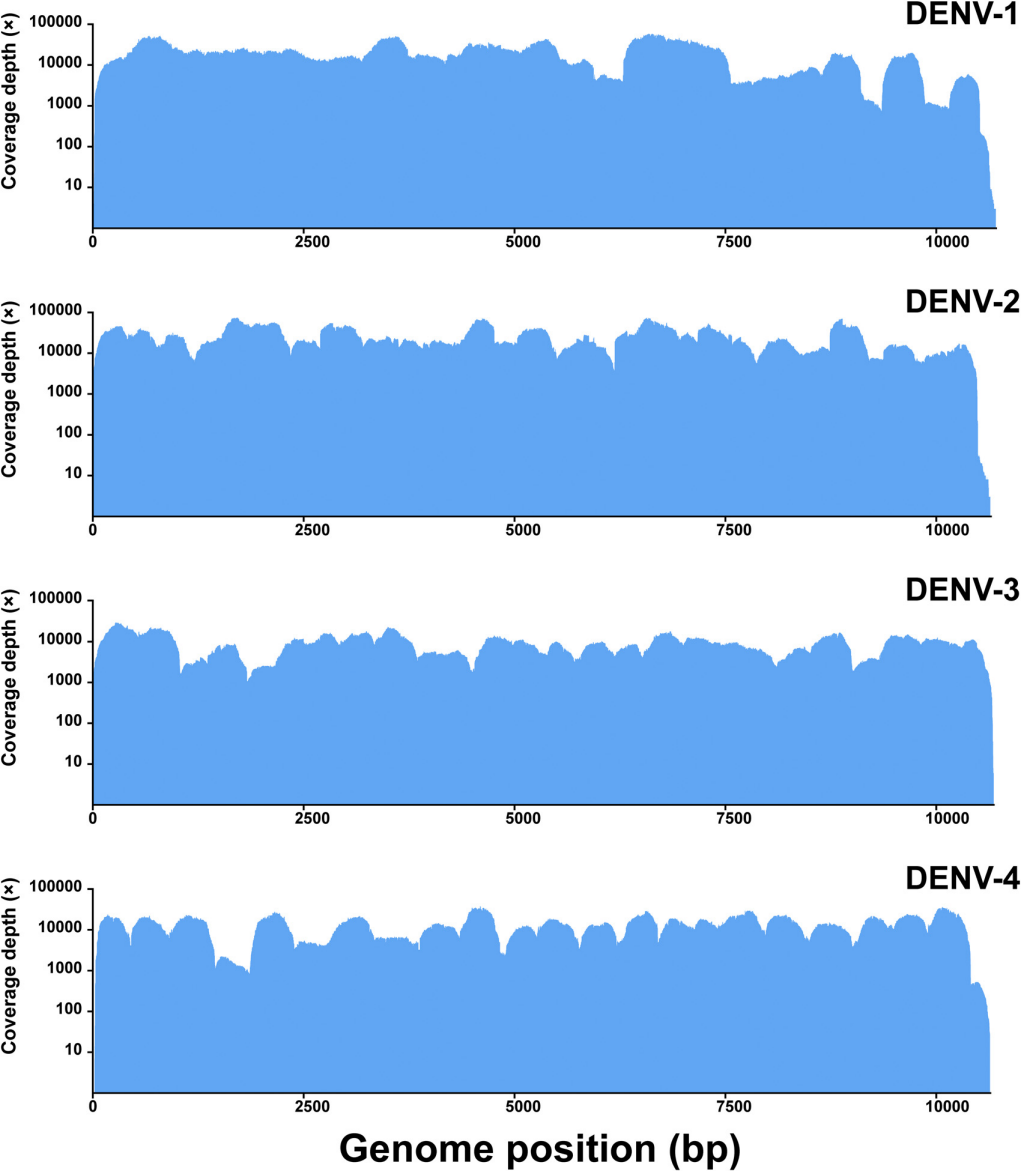
Sequencing coverage of DENV-positive RNA control samples. Downstream data generated using the Illumina MiniSeq platform were analyzed using CLC Genomics Workbench. Short (below 60 bp) and low-quality (below Q30) reads were discarded. The reference sequences used for mapping can be found under GenBank accession numbers NC_001477 (DENV-1), NC_001474 (DENV-2), NC_001475 (DENV-3), and NC_002640 (DENV-4).

**FIG 4:**
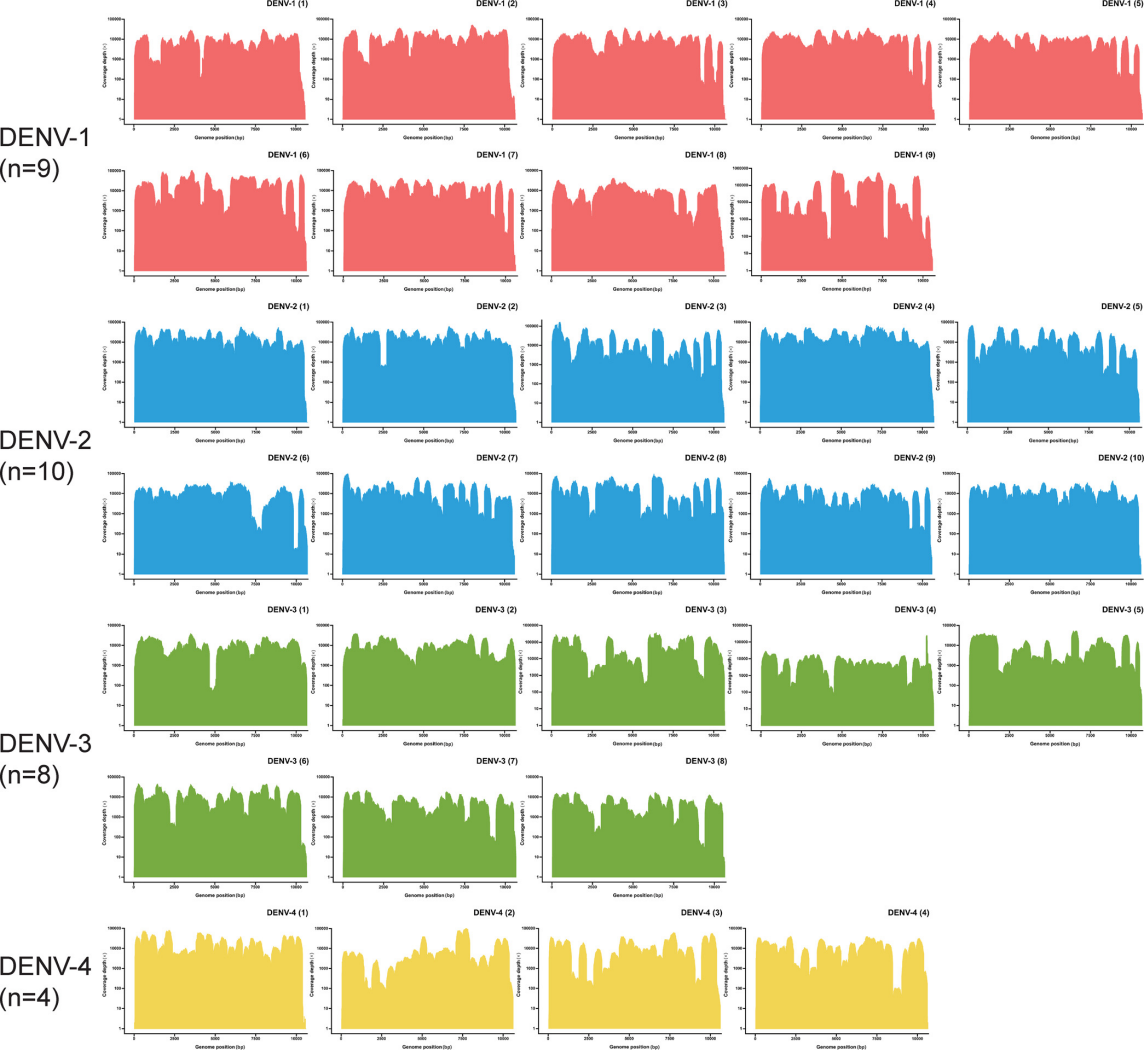
Coverage depth of the downstream data mapping results from the 31 DENV-positive clinical samples. Amplification efficiency varied across amplicons, genotypes, and serotypes of DENVs. The downstream data analysis was performed using CLC Genomics Workbench. The specific mapping results are shown in Table 1.

**FIG 5:**
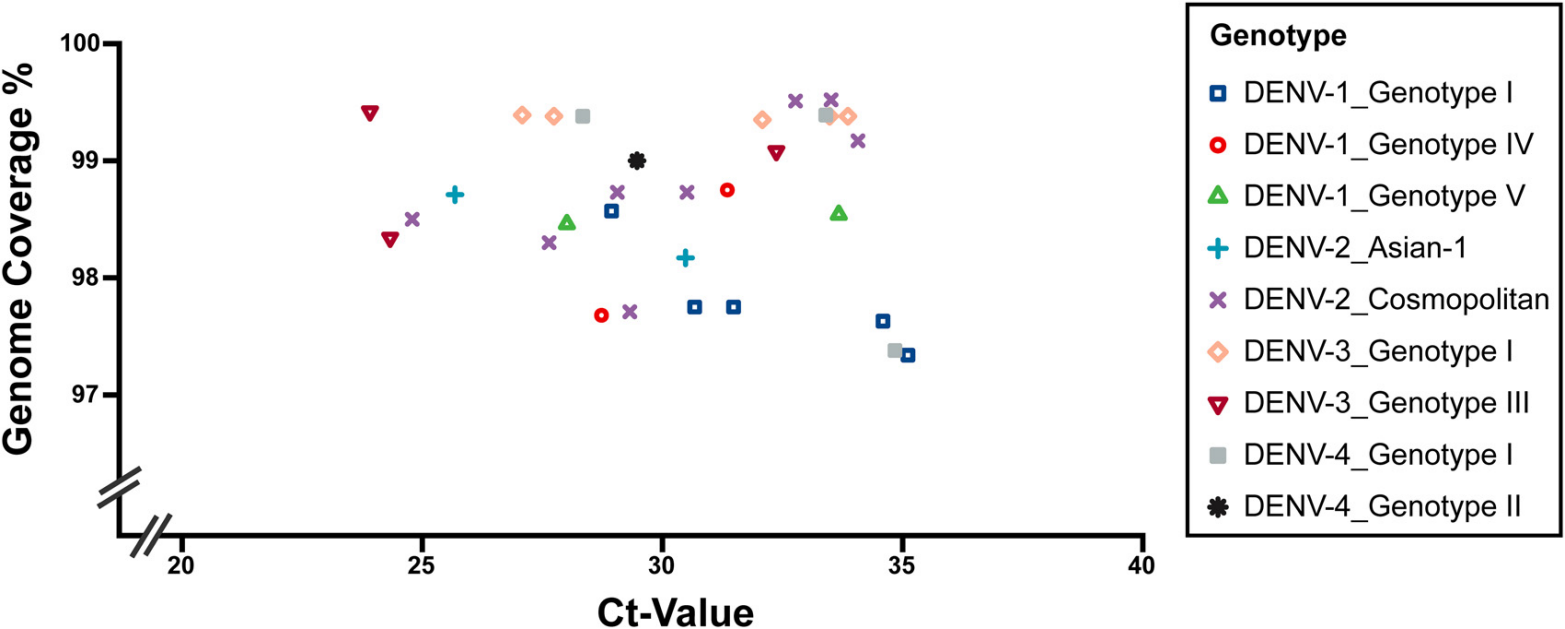
Genome coverage of the validation results for the 31 DENV-positive clinical samples. The downstream data were generated using the Illumina MiniSeq platform and analyzed using CLC Genomics Workbench. The consensus sequences were generated with a coverage depth of 20× or more with a process including primer trimming, mapping refinement, and manual checking to match the design length.

The average threshold cycle (*C_T_*) value of the 31 clinical samples was 30.38 (range, 23.91 to 35.11). Sequencing runs on the Illumina MiniSeq platform generated an average of 1,641,503.61 (range, 552,522 to 9,048,782) reads per sample. For each sample, 96.64% (range, 90.29% to 99.02%) of reads that passed the quality check (QC) on average mapped to the reference sequence. The consensus sequences generated at a coverage depth of 20× or more ranged in size from 10,370 to 10,672 bp (Fig. 3). The average lengths of DENV-1, 2, 3, and 4 were 10,525.89, 10,585.10, 10,623.00, and 10,534.25 bp, respectively. The average coverage of the whole genome was 98.66% (range, 97.34% to 99.52%), representing 100% coverage of the ORFs of the DENV whole genomes (Table 1; Fig. 4 and 5).

**TABLE 1:** Characteristics and resequencing results of the 31 serum samples used in this study

Sample no.	Serotype	Genotype	Origin	*C_T_* value	No. of multiplex PCR cycles^*a*^	Total no. of reads	No. (%) of reads passing QC	No. (%) of mapped reads	Consensus length (bp)^*b*^	Genome coverage %^*c*^	GenBank accession no.
1	DENV-1	Genotype I	Cambodia	34.59	35	724,516	711,176 (98.16)	703,038 (98.86)	10,481	97.63	ON908218
2	DENV-1	Genotype I	Cambodia	35.11	35	1,156,334	1,118,876 (96.76)	1,102,118 (98.5)	10,449	97.34	ON911333
3	DENV-1	Genotype I	China	31.48	35	903,552	879,180 (97.30)	861,728 (98.01)	10,493	97.75	ON908212
4	DENV-1	Genotype I	China	30.67	35	998,786	968,882 (97.01)	947,721 (97.82)	10,493	97.75	ON908213
5	DENV-1	Genotype I	Myanmar	28.94	30	716,166	696,108 (97.20)	682,316 (98.02)	10,582	98.57	ON908214
6	DENV-1	Genotype IV	Philippines	31.35	35	2,598,622	2,360,360 (90.83)	2,248,086 (95.24)	10,601	98.75	ON908215
7	DENV-1	Genotype IV	Philippines	28.73	30	1,446,840	1,317,216 (91.04)	1,304,321 (99.02)	10,486	97.68	ON908216
8	DENV-1	Genotype V	India	33.67	35	824,010	808,368 (98.10)	790,167 (97.75)	10,578	98.54	ON908220
9	DENV-1	Genotype V	Pakistan	28.01	30	9,048,782	8,720,706 (96.37)	8,373,768 (96.02)	10,570	98.46	ON908217
10	DENV-2	Asian-1	Cambodia	30.48	35	1,580,990	1,543,824 (97.65)	1,512,048 (97.94)	10,528	98.17	ON908224
11	DENV-2	Asian-1	Cambodia	25.68	30	1,654,768	1,609,702 (97.28)	1,592,716 (98.94)	10,586	98.71	ON908230
12	DENV-2	Cosmopolitan	Cambodia	30.51	35	1,864,624	1,824,360 (97.84)	1,762,892 (96.63)	10,588	98.73	ON908228
13	DENV-2	Cosmopolitan	China	33.51	35	1,864,958	1,836,084 (98.45)	1,809,751 (98.57)	10,672	99.52	ON908244
14	DENV-2	Cosmopolitan	China	29.06	30	1,248,968	1,204,208 (96.42)	1,175,097 (97.58)	10,588	98.73	ON908226
15	DENV-2	Cosmopolitan	China	32.77	35	1,088,398	1,054,456 (96.88)	1,039,000 (98.53)	10,671	99.51	ON908227
16	DENV-2	Cosmopolitan	China	24.79	30	1,418,676	1,378,876 (97.19)	1,350,961 (97.98)	10,563	98.50	ON908229
17	DENV-2	Cosmopolitan	Fuji	34.07	35	1,819,750	1,760,448 (96.74)	1,710,885 (97.18)	10,635	99.17	ON908222
18	DENV-2	Cosmopolitan	Niger	27.64	30	987,768	946,978 (95.87)	915,892 (96.72)	10,542	98.30	ON908223
19	DENV-2	Cosmopolitan	Philippines	29.32	30	1,147,268	1,124,084 (97.98)	1,101,247 (97.97)	10,478	97.71	ON908221
20	DENV-3	Genotype I	Bangladesh	27.74	30	934,478	913,328 (97.74)	907,556 (99.37)	10,641	99.38	ON908231
21	DENV-3	Genotype I	Bangladesh	33.48	35	983,134	939,924 (95.60)	925,211 (98.43)	10,641	99.38	ON908235
22	DENV-3	Genotype I	China	27.08	30	6,464,440	6,342,684 (98.12)	6,069,658 (95.70)	10,642	99.39	ON908245
23	DENV-3	Genotype I	China	32.08	35	1,169,398	1,145,242 (97.93)	806,195 (70.40)	10,637	99.35	ON908237
24	DENV-3	Genotype I	Thailand	33.86	35	943,922	926,944 (98.20)	922,406 (99.51)	10,641	99.38	ON908238
25	DENV-3	Genotype III	Ethiopia	32.37	35	1,093,332	1,072,608 (98.10)	1,069,631 (99.72)	10,608	99.08	ON908232
26	DENV-3	Genotype III	Maldives	23.91	30	635,608	573,900 (90.29)	563,987 (98.27)	10,645	99.42	ON908234
27	DENV-3	Genotype III	Maldives	24.33	30	552,522	502,510 (90.95)	495,078 (98.52)	10,529	98.34	ON908233
28	DENV-4	Genotype I	Myanmar	34.84	35	1,929,918	1,911,022 (99.02)	1,718,830 (89.94)	10,370	97.38	ON908246
29	DENV-4	Genotype I	Cambodia	33.40	35	899,420	886,368 (98.55)	877,240 (98.97)	10,584	99.39	ON908241
30	DENV-4	Genotype I	Cambodia	28.34	30	1,143,676	1,120,374 (97.96)	1,108,136 (98.91)	10,583	99.38	ON908242
31	DENV-4	Genotype II	Malaysia	29.47	30	1,042,988	1,026,594 (98.43)	1,023,079 (99.66)	10,600	99.00	ON908239

a The number of cycles set for multiplex PCR amplification depended on the *C_T_* values of the real-time RT-PCR: for samples with a *C_T_* value below 30, 30 cycles of amplification were used; for those with a *C_T_* value above 30, 35 cycles of amplification were used.

b The consensus sequences were generated with a coverage depth of 20× or more, using a primer-cutting process to match the design length.

c The whole-genome lengths of the reference sequences (GenBank accession numbers NC_001477, NC_001474, NC_001475, and NC_002640) were 10,735, 10,723, 10,707, and 10,649 bp, respectively.

The amplification efficiency varied across amplicons, different DENV serotypes/genotypes, and different samples with varied template concentrations, which led to different coverage depths of the mapping results (Table 1; Fig. 4 and 5). The consensus sequences of samples with a *C_T_* value of 30 or less (*n* = 14, with 30 amplification cycles) had an average whole-genome coverage of 98.68% (range, 97.68% to 99.42%). Those with a *C_T_* value higher than 30 (*n* = 17, with 35 amplification cycles) had an average whole-genome coverage of 98.64% (range, 97.34% to 99.52%). There were no significant differences in genome coverage between the two groups (*P* = 0.85).

All the consensus sequences generated using the NGS workflow matched the results previously obtained by Sanger sequencing, with a consistency level of >99.99% (Table S2). One base with a discrepancy between the NGS results and previously performed Sanger sequencing was found in sample number 17 (belonging to the DENV-2 cosmopolitan genotype) (Table 1). No more discrepancies were found in the NGS-Sanger comparison among the other 30 clinical samples (Table 1).

## DISCUSSION

WGS of pathogens plays a critical role in the rapid identification and investigation of the infection source during disease outbreaks, which calls for efficient, universal, and convenient tools with less complexity. In this study, we designed 4 sets of primer panels for each serotype of DENV and validated the panels with 31 DENV-positive serum samples (*C_T_* value range, 23.91 to 35.11). Each of the serotype-specific panels was validated with serum samples belonging to 2 or 3 genotypes within the serotype. The length of consensus sequences generated with a coverage depth of 20× ranged from 10,370 to 10,672 bp, with 100.00% coverage of the ORF and 97.34% to 99.52% coverage of the whole genome. The amplification efficiency varied across amplicons, genotypes, and serotypes of DENVs. Additionally, the primer panels we used in this study are adaptable to other sequencing platforms, if required (24, 25).

Complete or partial genome sequencing has been used in DENV surveillance. Historically, segmental reverse transcription-PCR (RT-PCR) combined with Sanger DNA sequencing of the DENV E gene (about 1,600 bp to 1,800 bp) was successfully applied (10, 30). With the requirement of accuracy in molecular epidemiological studies, WGS of DENV has been gradually applied (22, 25, 31–34). NGS technologies have recently enabled large-scale surveillance of infectious diseases, and the power and utility of NGS are based on its massively parallel interrogation of nucleic acids. Parallel segmental RT-PCR with overlapping amplicons for Sanger sequencing or NGS platforms has been developed for DENV WGS, which often requires virus isolation and purification before RNA extraction, reverse transcription, and PCR amplification.

Primal Scheme is a useful and convenient online tool for primer design, providing a complete pipeline for the development of efficient multiplex primer schemes (24). Users have only to select the appropriate references and set a desired PCR amplicon length on the website, and then the online tool will do the rest of the work and return the set of primers and their parameters, greatly shortening the time required for primer design. In this study, we used two-pooled, serotype-specific panels with 23 to 29 pairs of primers, amplifying products about 500 bp long with about 100 to 200 bp overlap for a viral genome about 10,700 bp long. The primer panels that we finally set up after rounds of computational mapping, screening, and manual modifications were generated using Primal Scheme at first. When a mutant occurred on the primer binding site, the rational use of degenerate bases could effectively keep the primer panels working; self-dimers, cross-dimers, hairpin structures with high *T_m_*, and mismatches could be avoided by manual modification at the 5′ end of the primers. Sometimes, hairpin *T_m_* could be greatly reduced by replacing one or more bases at the key position of the hairpin structure. It is important to note that computational mapping, screening, and manual checkup and modification of the primers are essential for success.

Sequencing directly from clinical samples is faster, less laborious, less time-consuming, and more flexible (24). However, clinical samples for DENV detection can vary in both RNA quality and viral load, making genome recovery unreliable. Challenges with samples having low RNA concentrations (*C_T_* value, ≥35) and/or degraded RNA templates have been one of the difficulties in the WGS of DENV (35). For WGS of SARS-CoV-2, the multiplex PCR strategy is effective at generating whole-genome sequences up to a *C_T_* value of 33, and “singleplex” nested PCR is effective up to a *C_T_* value of 35, varying based on the sample quality (36). The generation of multiplex overlapping amplicons can be complicated by the template concentration, sequence diversity, primer specificity, and PCR amplification efficiency (26, 27, 36–38). Samples with low RNA concentrations and/or degraded RNA templates are usually unsuited for long-distance amplification. Reducing the sizes of amplicons and increasing the number of primers, or optionally using nested PCR, may be useful for samples with low RNA concentrations and/or degraded RNA templates when amplification of longer products fails, which may also result from the operational complexity in target enrichment and library preparation. The multiplex PCR strategy offers an efficient, cost-effective, scalable system and adds little time and complexity as sample numbers increase. The sequencing cost per sample on the Illumina and Nanopore platforms decreases with increasing numbers of samples, and the turnaround time remains the same (36).

To validate the universality of the primer panels, we used DENV-positive clinical samples belonging to as many different serotypes and genotypes as possible. The amplification cycles for each sample were carefully set up. The amplification efficiency varied among amplicons, so that the homogeneity of the downstream data would be affected when the number of cycles for multiplex PCR was set too high. It is important to note that in order to achieve a balance between the amplification efficiency and homogeneity of the downstream data, the number of amplification cycles should be set carefully to match the *C_T_* value of each sample (we recommend 30 cycles for those samples with a *C_T_* value below 30 and 35 cycles for those samples with a *C_T_* value above 30), which also increases the possibility of successful mapping. The results of the validation using 31 clinical samples (17 with a *C_T_* value higher than 30) were acceptable, and all of the samples had 100% coverage of the DENV genome ORF. The mapping percentage of the downstream data ranged from 86.10% to 99.58%, which indicated that the flow cell and data had been fully utilized. Furthermore, there is a complementary method called metagenomic sequencing with spiked primer enrichment (MSSPE) with less biased amplification, less sample cross-contamination, and the ability to increase the yield of viral reads for detection and genome recovery, which can be used in combination with multiplex PCR amplification (23).

DENVs are known for their genetic diversity within serotype groups and geographical regions (1, 16). Currently, consolidation and urban circulation of DENV are rare in mainland China (17, 39–43). Due to the abundance of rainfall in summer and the wide distribution of Aedes albopictus, DENV outbreaks in mainland China are usually caused by imported strains. The increasing frequency of international exchange has complicated outbreak tracing. Therefore, the primer panels we designed focused on universality. The references selected in the design stage and the clinical samples used in the validation stage both covered as many genotypes as possible, all over the world. Thus, the panels will be useful in future local DENV outbreaks. Furthermore, DENV population sequencing by NGS can increase our understanding of the changing epidemiology and evolution of the DENV genome at the molecular level, which demands universal primer panels and combination with NGS platforms.

Our study has limitations. One round of real-time RT-PCR for serotyping at the beginning of the entire workflow is essential because of the lack of universality among the serotypes. Moreover, cross-contamination is a serious potential problem when working with amplicon sequencing. Targeted NGS methods are highly sensitive to amplicon contamination from previous experiments. Extreme caution should be taken to keep laboratories and their environments free of amplicon contamination.

### Conclusion.

A serotype-specific WGS method for DENVs was described and validated with 31 DENV-positive clinical samples belonging to different serotypes and genotypes of DENV. Additionally, details of the computational simulation, mapping, screening, and manual modification during the primer design stage were described. For each DENV serotype, we designed a serotype-specific primer panel, which included two pools of primers for the WGS. The universality and the amplification efficiency were confirmed from the mapping results of 31 samples, especially those with low RNA concentration and/or degraded RNA templates. The mapping results matched the consensus sequences previously determined and assembled using the Sanger method. This study provides a universal, rapid, and effective tool for the integration of genomics with dengue surveillance. However, the panels should be validated with more clinical samples, especially for those genotypes not involved in this study, and the primer panels should be continuously optimized to match future mutants of DENV.

## MATERIALS AND METHODS

### Summary of the entire workflow.

**FIG 6:**
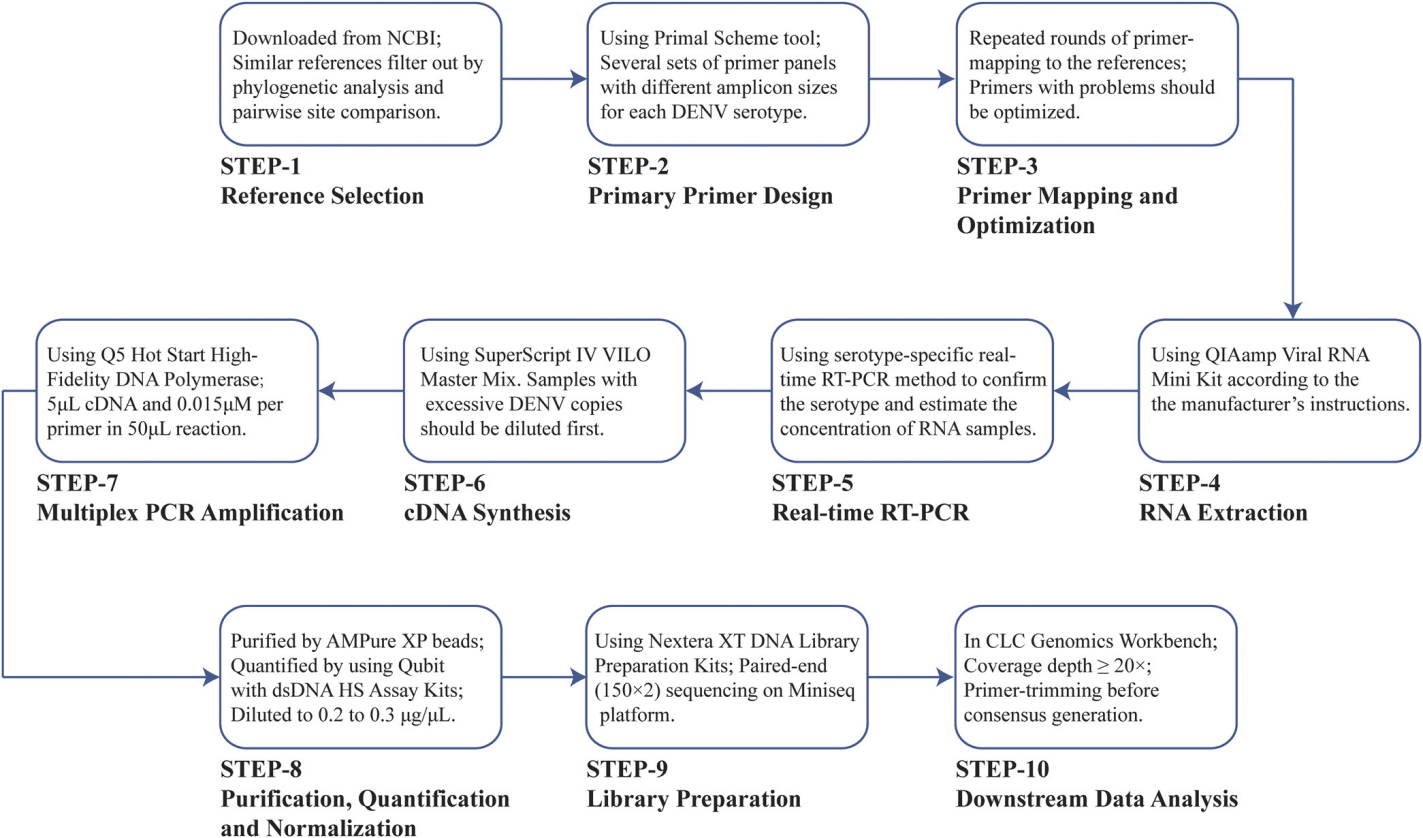
Complete 10-step workflow in this study and the key points in each step.

The complete workflow in this study is shown in detail in Fig. 6, including (i) reference sequences selection; (ii) primary primer design; (iii) primer mapping, optimization, and pooling; (iv) RNA extraction; (v) real-time RT-PCR for concentration estimation and serotyping; (vi) cDNA synthesis; (vii) multiplex PCR amplification; (viii) product purification, quantification, and normalization; (ix) library preparation; and (x) downstream data analysis.

### Primer design.

The DENV primary reference sequences were downloaded from the National Center for Biotechnology Information (NCBI) database, covering all genotypes of all DENV serotypes (DENV-1 to DENV-4) circulating on every endemic continent. After alignment using the Clustal-W method, we used phylogenetic analysis and pairwise site comparison to filter out similar references for each serotype. Finally, 110 DENV references (26 DENV-1, 33 DENV-2, 31 DENV-3, and 20 DENV-4; see Table S3 in the supplemental material) were selected for the primer design. A maximum likelihood phylogenetic analysis of each DENV serotype was performed using the online tool PhyML 3.0 (http://www.atgc-montpellier.fr/phyml/) with an automatic nucleotide substitution selection model within Smart Model Selection (SMS; http://www.atgc-montpellier.fr/sms/). The results are shown in Fig. S1; the reference sequences we used for the primer design are marked with an asterisk (44, 45).

The Primal Scheme online tool was used to design the primary primer panels. First, we set the range for the imbricated amplicon sizes to about 400, 450, and 500 bp. Several sets of primer panels for each serotype with different amplicon sizes were returned using the program. To maximize the sequencing coverage percentage, we preferentially mapped primer panels with 500-bp amplicons to the references of each respective serotype. According to the mapping results, primers with too many mismatches or low specificity were manually modified, removed, or redesigned. Additionally, self-dimer and hairpin structures with a melting temperature (*T_m_*) of >50°C were avoided. Repeated rounds of primer mapping were performed to ensure primer panels with theoretically acceptable performance in the computational simulation. Then, we divided the selected primer panel of each serotype into two primer pools for 2 tubes of PCR amplification separately, i.e., odd-numbered and even-numbered primers were divided into primer pool 1 and pool 2, respectively. All the primers were synthesized by Sangon Biotech Co., Ltd.

### RNA control and clinical samples for validation.

DENV strains previously isolated at the Guangzhou Center for Disease Control and Prevention (GZCDC) from 2017 to 2019 (GenBank accession numbers ON908243, ON908225, ON908236, and ON908240) were used as the RNA control in the primary assessment of primer panels. Additionally, a total of 31 DENV-positive serum samples collected by GZCDC between 2017 and 2021 were resequenced for validation of the primer panels in this study. Among the 31 samples, 9 were DENV-1 positive, 10 were DENV-2 positive, 8 were DENV-3 positive, and 4 were DENV-4 positive. The DENV genome sequences previously determined using Sanger dideoxynucleotide sequencing are available at GenBank (Table 1; Table S2).

### RNA extraction.

Nucleic acids were extracted from the serum samples and DENV isolates using the Qiagen viral RNA minikit (number 52906) according to the manufacturer’s instructions.

### Real-time RT-PCR.

We used a real-time RT-PCR method with TaqMan probes to estimate the concentration of target templates and confirm the DENV serotype of the RNA samples. The real-time RT-PCR was performed using the One Step PrimeScript RT-PCR kit (Perfect Real Time, TaKaRa; number RR064A) with DENV-specific primers and probes (listed in Table S4) recommended by the National Health Commission of the People’s Republic of China (46).

### cDNA synthesis.

RNA samples with a *C_T_* value below 20 were diluted 10- to 100-fold before cDNA synthesis. The cDNA synthesis was performed using SuperScript IV VILO master mix (Invitrogen; number 11756500), according to the manufacturer’s instructions.

### Multiplex PCR amplification.

The primer panel selected for each serotype was previously divided into two primer pools. For each sample, 2 PCR amplification tubes, one with primer pool 1 and one with primer pool 2, were required. The primers from pools 1 and 2 were each diluted into 100 μM and mixed well, according to the presets mentioned above. We chose Q5 Hot Start high-fidelity DNA polymerase (NEB; number M0493L) for the multiplex PCR amplifications. Amplicons were generated in a 50-μL reaction for each pool containing 5 μL of cDNA templates, with the concentration of primers set at 0.015 μM per primer. The multiplex PCR was performed as follows: hot start at 98°C for 30 s, followed by 30 or 35 cycles (depending on the *C_T_* value of the sample; we recommend 30 cycles for samples with a *C_T_* value below 30 and 35 cycles for samples with a *C_T_* value above 30) of 98°C for 10 s, 50°C for 15 s, and 72°C for 5 min, and a final extension at 72°C for 10 min.

### Product purification, quantification, and normalization.

The PCR products of pools 1 and 2 from the same sample were mixed into one tube before being purified using AMPure XP beads (Beckman Coulter; number A63881) according to the manufacturer’s instructions. The purified DNA samples were quantified using a Qubit 4.0 fluorometer (Invitrogen; number Q33238) with Qubit double-stranded DNA (dsDNA) high-sensitivity (HS) assay kits (Invitrogen; number Q32851). According to the quantification results, we diluted the DNA samples to 0.2 to 0.3 μg/μL prior to library preparation.

### Library preparation and NGS sequencing.

Briefly, DNA libraries were constructed using Nextera XT DNA library preparation kits (Illumina; number FC-131-1024), according to the manufacturer’s instructions. After enzymatic fragmentation, library amplification was performed for the fragmented samples with combinatorial dual indexes (Nextera DNA indexes, Illumina; number FC-121-1011). Library quantification was conducted using a Qubit 4.0 fluorometer (Invitrogen; number Q33238) with Qubit dsDNA HS assay kits (Invitrogen; number Q32851) again. The barcoded libraries were pooled with equal concentrations and diluted to 1.8 pM before sequencing on the MiniSeq platform (Illumina) with the 300-cycle mild output reagent cartridge (Illumina; number FS-420-1004).

### Data analysis.

The downstream NGS data were analyzed using CLC Genomics Workbench (Qiagen; number 832021). The bioinformatics workflow for the downstream data analysis was performed using CLC Genomics Workbench as follows: (i) data QC, (ii) read trimming, (iii) primary mapping, (iv) primer trimming from mapping, (v) mapping refining, (vi) consensus extraction, and (vii) manual checking and correction. Short (below 60 bp) and low-quality (below Q30) reads were discarded. The genomes submitted under GenBank accession numbers NC_001477 (DENV-1), NC_001474 (DENV-2), NC_001475 (DENV-3), and NC_002640 (DENV-4) were used as the reference sequences for mapping. The consensus sequences were generated with a coverage depth of 20× or more, using a primer-trimming process to match the design length. The genome coverage, coverage depth, and consistency of the consensus sequences were calculated for evaluation.

### Statistical analysis and data visualization.

Statistical analysis and data visualization in this study were conducted using Hiplot (https://hiplot.org), a Web service for facilitating the visualization of biomedical data (47).

### Ethics.

This study was approved by the Ethics Committee of the Center for Disease Control and Prevention (CDC) of Guangzhou (GZCDC-ECHR-2022A0001). Written informed consent was obtained from patients regarding surveillance and data related to disease control and analysis. All information related to individuals in this study was pseudonymized.

### Data availability.

The data that support the findings of this study have been deposited at the CNGB Sequence Archive (CNSA) of the China National GeneBank DataBase (CNGBdb) under accession numbers CNP0003264 and CNP0003265 for the downstream data of the clinical samples and the RNA controls, respectively (48, 49). All the DENV strains in our collection have been deposited at the China National GeneBank, a nonprofit, public service-oriented organization in China.
