# Supplementary material for: A Serotype-Specific and Multiplex PCR Method for Whole-Genome Sequencing of Dengue Virus Directly from Clinical Samples
Source: Microbiol Spectr. 2022 Sep 12;10(5):e01210-22. doi: 10.1128/spectrum.01210-22 (PMC9602986; doi:10.1128/spectrum.01210-22)
Supplement: Supplemental file 1 — Fig. S1 and Tables S1-S4. Download spectrum.01210-22-s0001.pdf, PDF file, 1.1 MB [file spectrum.01210-22-s0001.pdf]

A

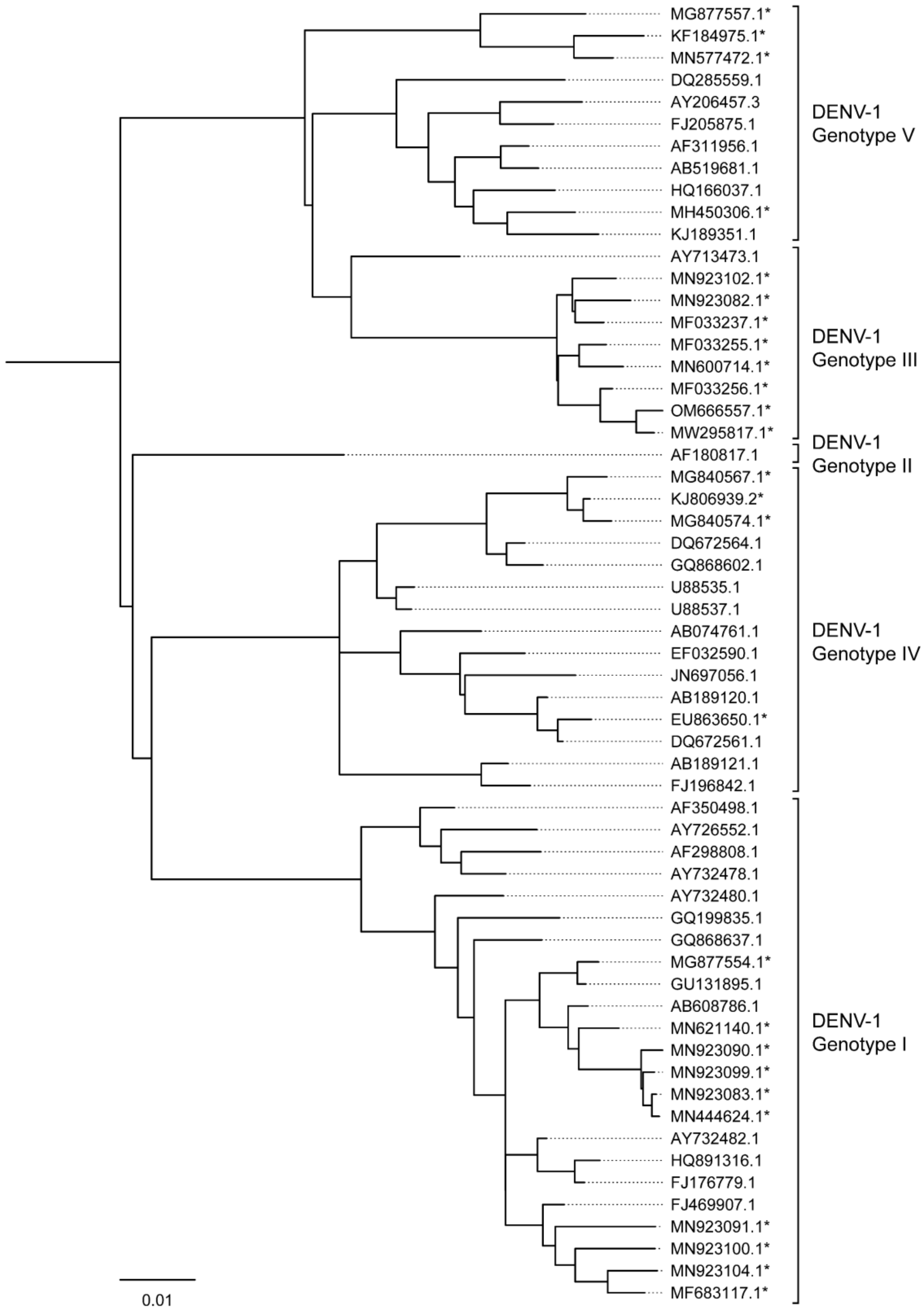
MG877557.1*
KF184975.1*
MN577472.1*
DQ285559.1
AY206457.3
FJ205875.1
AF311956.1
AB519681.1
HQ166037.1
MH450306.1*
KJ189351.1
DENV-1
Genotype V
AY713473.1
MN923102.1*
MN923082.1*
MF033237.1*
MF033255.1*
MN600714.1*
MF033256.1*
OM666557.1*
MW295817.1*
DENV-1
Genotype III
AF180817.1
DENV-1
Genotype II
MG840567.1*
KJ806939.2*
MG840574.1*
DQ672564.1
GQ868602.1
U88535.1
U88537.1
AB074761.1
EF032590.1
JN697056.1
AB189120.1
EU863650.1*
DQ672561.1
AB189121.1
FJ196842.1
DENV-1
Genotype IV
AF350498.1
AY726552.1
AF298808.1
AY732478.1
AY732480.1
GQ199835.1
GQ868637.1
MG877554.1*
GU131895.1
AB608786.1
MN621140.1*
MN923090.1*
MN923099.1*
MN923083.1*
MN444624.1*
AY732482.1
HQ891316.1
FJ176779.1
FJ469907.1
MN923091.1*
MN923100.1*
MN923104.1*
MF683117.1*
DENV-1
Genotype I
0.01

B

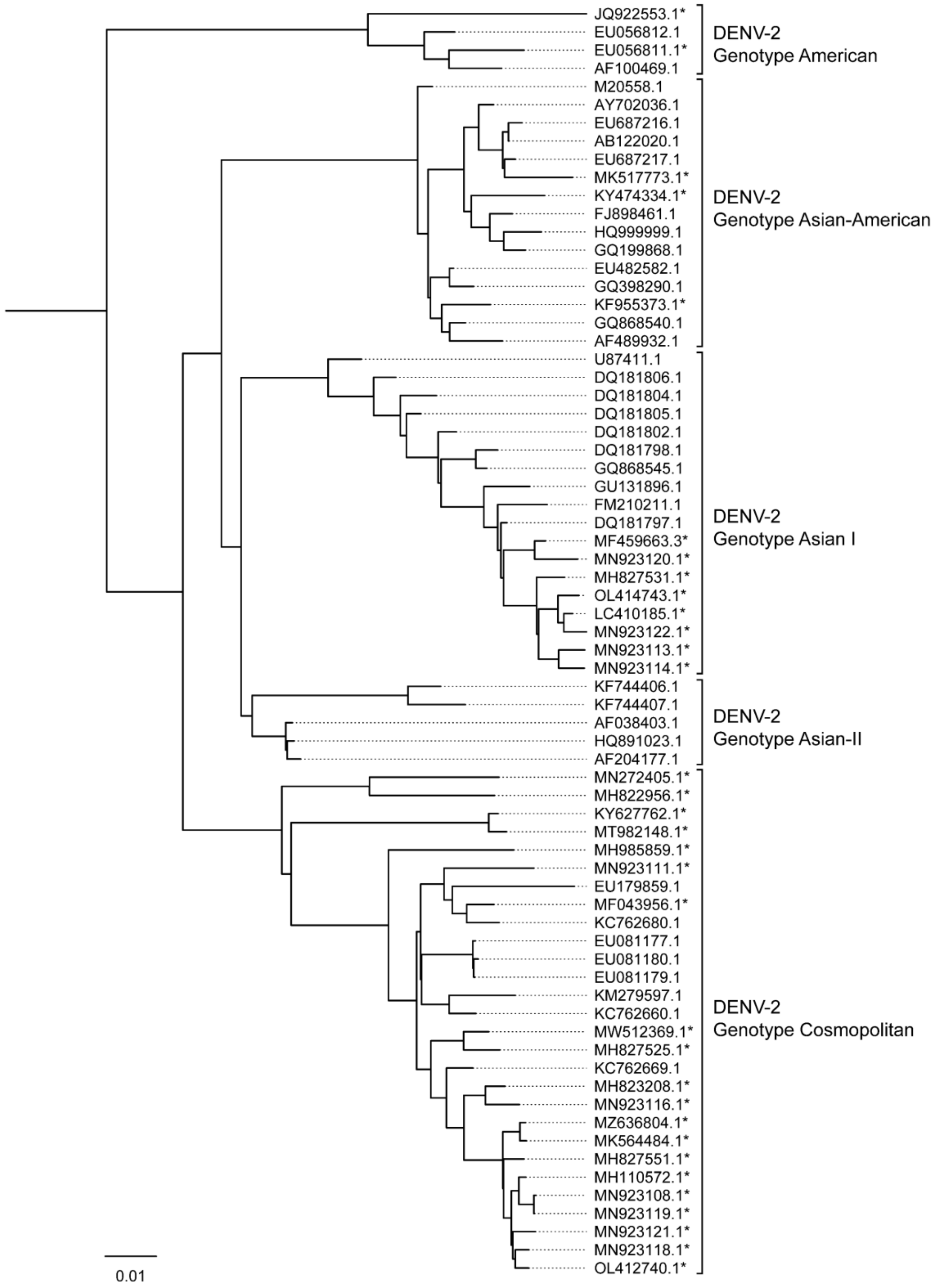
JQ922553.1*
EU056812.1
EU056811.1*
AF100469.1
DENV-2
Genotype American
M20558.1
AY702036.1
EU687216.1
AB122020.1
EU687217.1
MK517773.1*
KY474334.1*
FJ898461.1
HQ999999.1
GQ199868.1
EU482582.1
GQ398290.1
KF955373.1*
GQ868540.1
AF489932.1
DENV-2
Genotype Asian-American
U87411.1
DQ181806.1
DQ181804.1
DQ181805.1
DQ181802.1
DQ181798.1
GQ868545.1
GU131896.1
FM210211.1
DQ181797.1
MF459663.3*
MN923120.1*
MH827531.1*
OL414743.1*
LC410185.1*
MN923122.1*
MN923113.1*
MN923114.1*
DENV-2
Genotype Asian I
KF744406.1
KF744407.1
AF038403.1
HQ891023.1
AF204177.1
DENV-2
Genotype Asian-II
MN272405.1*
MH822956.1*
KY627762.1*
MT982148.1*
MH985859.1*
MN923111.1*
EU179859.1
MF043956.1*
KC762680.1
EU081177.1
EU081180.1
EU081179.1
KM279597.1
KC762660.1
MW512369.1*
MH827525.1*
KC762669.1
MH823208.1*
MN923116.1*
MZ636804.1*
MK564484.1*
MH827551.1*
MH110572.1*
MN923108.1*
MN923119.1*
MN923121.1*
MN923118.1*
OL412740.1*
DENV-2
Genotype Cosmopolitan
0.01

C

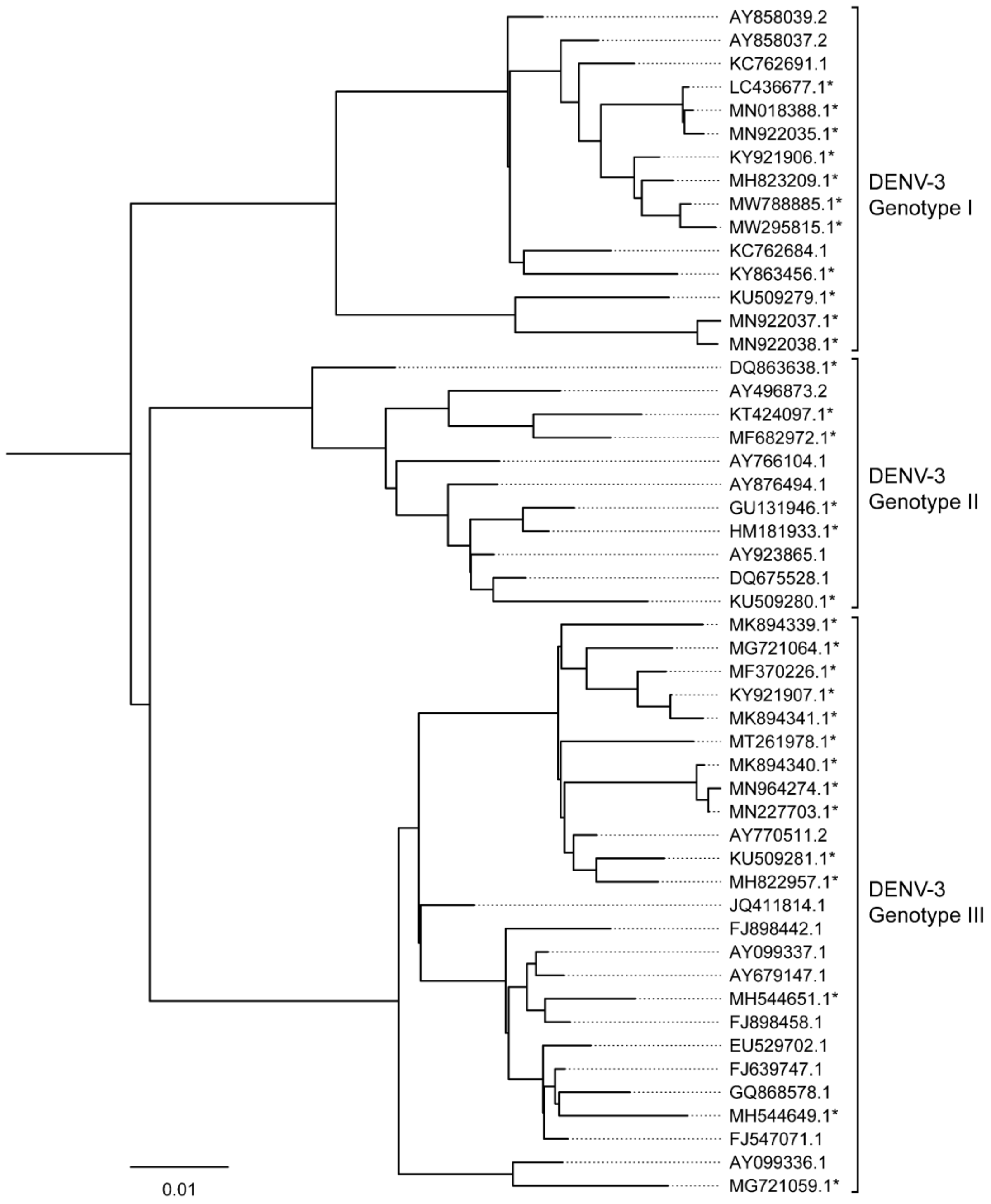
AY858039.2
AY858037.2
KC762691.1
LC436677.1*
MN018388.1*
MN922035.1*
KY921906.1*
MH823209.1*
MW788885.1*
MW295815.1*
KC762684.1
KY863456.1*
KU509279.1*
MN922037.1*
MN922038.1*
DENV-3 Genotype I
DQ863638.1*
AY496873.2
KT424097.1*
MF682972.1*
AY766104.1
AY876494.1
GU131946.1*
HM181933.1*
AY923865.1
DQ675528.1
KU509280.1*
DENV-3 Genotype II
MK894339.1*
MG721064.1*
MF370226.1*
KY921907.1*
MK894341.1*
MT261978.1*
MK894340.1*
MN964274.1*
MN227703.1*
AY770511.2
KU509281.1*
MH822957.1*
JQ411814.1
FJ898442.1
AY099337.1
AY679147.1
MH544651.1*
FJ898458.1
EU529702.1
FJ639747.1
GQ868578.1
MH544649.1*
FJ547071.1
AY099336.1
MG721059.1*
DENV-3 Genotype III
0.01

D

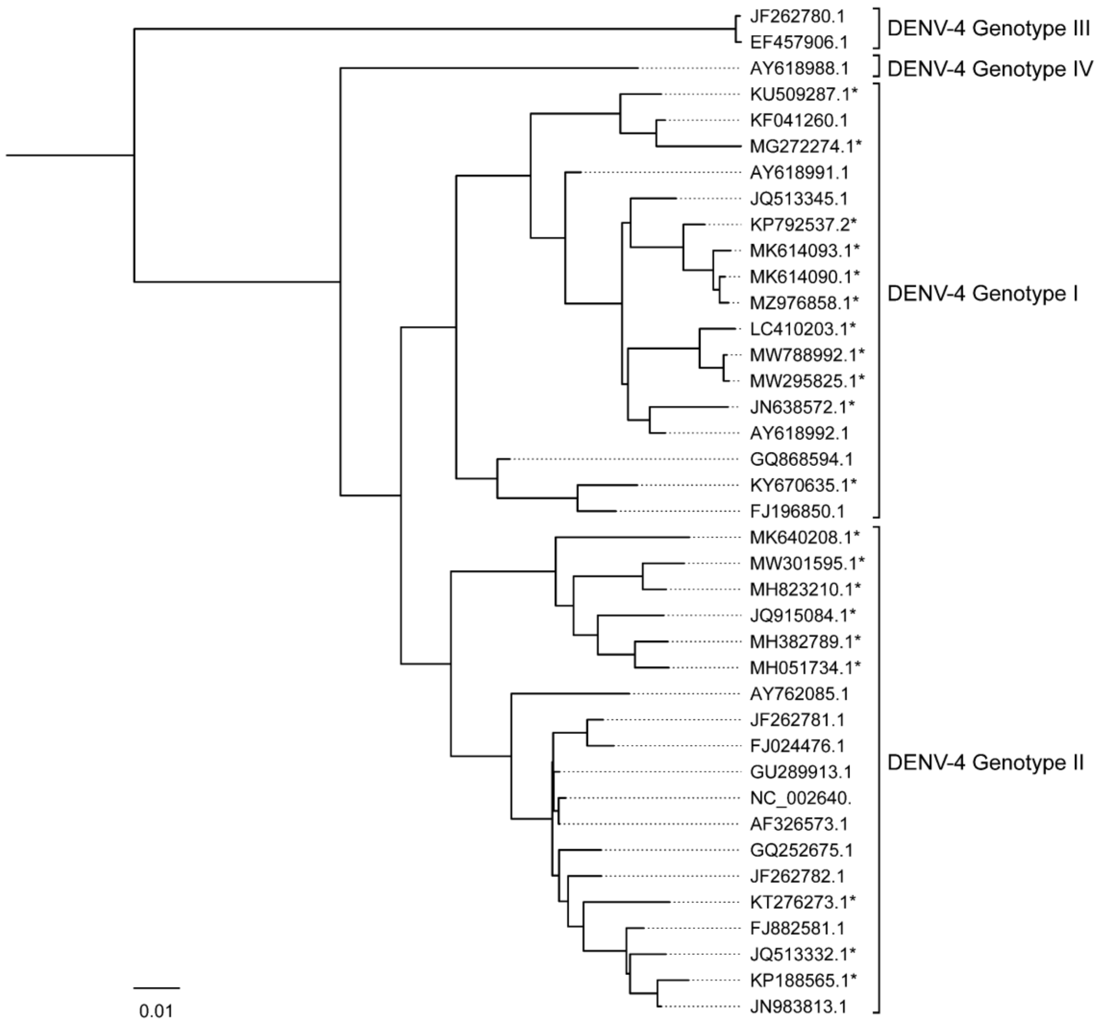

Figure-S1 The maximum-likelihood phylogenetic analysis of (A) DENV-1, (B) DENV-2, (C) DENV-3 and (D) DENV-4 by PhyML 3.0. The nucleotides substitution model for each serotype was automatic selected by Smart Model Selection. The references used for the designing of primer panels were marked with “*”.

Table S1. The primer panels designed for the whole genome sequencing of DENV in this study.

Name [a]	Direction	Sequence (5'→3')	Position [b]
DENV-1_1F	Forward	GTCTACGTGGACCGACAAGAAC	9-30
DENV-1_1R	Reverse	AGATCTCTTTCTTGAAACCYCGT	337-359
DENV-1_2F	Forward	CAGTTGGCGAAGAGATTCTCA	176-196
DENV-1_2R	Reverse	ACATAACTCTCCCAAATCCATYG	549-571
DENV-1_3F	Forward	GGAGAGCCACACATGATAGT	458-477
DENV-1_3R	Reverse	CCTATTCCCACGCATCGCAT	935-954
DENV-1_4F	Forward	TGATAGCCTTTTTTTTAGCACATGC	840-864
DENV-1_4R	Reverse	GTTTTCATATTGAACTATCTTTCCTTC	1310-1336
DENV-1_5F	Forward	ACGAACGTTTGTGGAYAGAGG	1213-1233
DENV-1_5R	Reverse	TTCTTGTGATCCCARTACRACYA	1683-1705
DENV-1_6F	Forward	TTTGCTAGTCACATTYAAGACAG	1639-1661
DENV-1_6R	Reverse	TGCTTCCTTTCTTRAACCAGCT	2102-2123
DENV-1_7F	Forward	TACTGACAAAGAAAAACCAGTCAAYATT	2008-2035
DENV-1_7R	Reverse	ATTAGTGACAAAAATGCCRCTTCCA	2464-2488
DENV-1_8F	Forward	CTRGTMACACTRTACCTAGGAG	2384-2405
DENV-1_8R	Reverse	TTTCAGCCATATGTTTGTYGTGA	2907-2929
DENV-1_9F	Forward	TTGAGACGACTACCTTCATYATYGA	2802-2826
DENV-1_9R	Reverse	GAGATGGTCCTCGATTTCCACA	3290-3311
DENV-1_10F	Forward	ACAGACCAGGATATTTCACACAAAC	3186-3210
DENV-1_10R	Reverse	CAACAGCAAACATYGGTCTCAT	3722-3743
DENV-1_11F	Forward	TATAATGGGACAAYTGACRTGGA	3604-3626
DENV-1_11R	Reverse	AACATGGTTAGTGGTTTRCATCC	4073-4095
DENV-1_12F	Forward	ATCATGATGTTAAAATTGTTGACYGA	3866-3891
DENV-1_12R	Reverse	GACCTCCACTAAGATRTTGTGT	4342-4363
DENV-1_13F	Forward	TCCTGGGAAGAAGAAGCAGA	4307-4326
DENV-1_13R	Reverse	CTCCAACCTCCTCCATATGAGA	4749-4770
DENV-1_14F	Forward	AGGGGAGCTGTCCTYATGTA	4679-4698
DENV-1_14R	Reverse	CTTTTTATGGCCTCACGRACTAT	5141-5163
DENV-1_15F	Forward	ATTGAGGACGARGTGTTYARGA	5054-5075
DENV-1_15R	Reverse	TCAGGAATGTCTCTTTCCTCATCTT	5520-5544
DENV-1_16F	Forward	AATCTTTATGACRGCYACYCC	5455-5475
DENV-1_16R	Reverse	TCGTTGTTTAAAGGCTGTCCCA	5946-5967
DENV-1_17F	Forward	GCAGAGGAAGAATTGGAAGGAAC	5892-5914
DENV-1_17R	Reverse	TAGATCACCTGAGACRCTTCT	6374-6394
DENV-1_18F	Forward	GAAGGAGAAAGAAAGAAACTRCGACC	6275-6300
DENV-1_18R	Reverse	CATGAATAACAAACCTATCACCAC	6785-6808
DENV-1_19F	Forward	AGTGTGGARCCCCATTGGAT	6671-6690
DENV-1_19R	Reverse	TTCTCTAGTACCTTTTGCTTGCA	7203-7225
DENV-1_20F	Forward	GGATGGCCAATATCRAARATGGA	7070-7092
DENV-1_20R	Reverse	CTACCTCCTCCTAAAGATTTCAT	7547-7569
DENV-1_21F	Forward	GGAAAATTCTGGAACACYACGAT	7457-7479
DENV-1_21R	Reverse	GCCATTGGATTAGGTTCYTCRT	7902-7923

DENV-1_22F	Forward	GAAGAGGTGGCTGGTCATAY	7821-7840
DENV-1_22R	Reverse	GTCCACGTCTCTTTCATAYGTTGG	8318-8341
DENV-1_23F	Forward	TCAGCAGTAAATATGACATCYAGAAT	8258-8283
DENV-1_23R	Reverse	ACTTGGCTGTTACCTCCATRAT	8687-8708
DENV-1_24F	Forward	ACAGAGGGTGTTTAAAGAGAAAGT	8623-8646
DENV-1_24R	Reverse	TCCAAGTTTGTGGAGTCCYTCTC	9096-9118
DENV-1_25F	Forward	GGAAGTCGYGCAATATGGTACAT	8978-9000
DENV-1_25R	Reverse	CAATTCGCTRGGTGAAAAGATTC	9450-9472
DENV-1_26F	Forward	TCATATCCAGACGTGACCAGA	9345-9365
DENV-1_26R	Reverse	TGGAAATACATCAGCTGCCACAT	9851-9873
DENV-1_27F	Forward	ATGCCGCAAYCAAGATGAACT	9751-9771
DENV-1_27R	Reverse	TCACTCTCGTTYTTGAATCTYTTCA	10227-10251
DENV-1_28F	Forward	CCACCAACATACAAGTRGCYATA	10149-10171
DENV-1_28R	Reverse	CTCCACTAACCACTAGTCTGCTA	10503-10525
DENV-1_29F	Forward	TCCAAGGACGTWAAATGAAGTCAG	10379-10402
DENV-1_29R	Reverse	GATTCAACRGCAYCATTCCAT	10705-10725
DENV-2_1F	Forward	AGTTGTTAGTCTACGTGGACC	1-21
DENV-2_1R	Reverse	TACTGACGATCATGTGTGGTTC	463-484
DENV-2_2F	Forward	GCATAATTATCATGCTGATYCCAACA	404-429
DENV-2_2R	Reverse	ATTATGGTAAAGCCTGGATGTCT	823-845
DENV-2_3F	Forward	AACTGAAACATGGATGTCATCAGA	756-779
DENV-2_3R	Reverse	TCTACCATGGAGTGTTTGCAGA	1208-1229
DENV-2_4F	Forward	ATAGAATCCCGTTGCCCAACA	1144-1164
DENV-2_4R	Reverse	CAATGTCTCTTTCTGTATCCARTTTG	1622-1647
DENV-2_5F	Forward	AGATGGTGTTGCTGCARATGGA	1520-1541
DENV-2_5R	Reverse	ATTGTTCCATGTTGTGTTTCTGCTA	1871-1895
DENV-2_6F	Forward	CTTAAGTGCAGGCTGAGAATG	1783-1803
DENV-2_6R	Reverse	TGCGTGAGTTCATTCCTATCCA	2329-2350
DENV-2_7F	Forward	ATTCAGTGGGGTTTCATGGACY	2277-2298
DENV-2_7R	Reverse	TTCCAAGAGTACTTCARCTCAGT	2746-2768
DENV-2_8F	Forward	ACAGGAGACATYAAAGGWATCATG	2689-2712
DENV-2_8R	Reverse	TGTTTGTGTGTAATAGCCTGGTC	3191-3213
DENV-2_9F	Forward	GCAATGGAGTTCTAGAAAGYGAGAT	3119-3143
DENV-2_9R	Reverse	CCAAATCTCGAAAGGACATRTTC	3615-3637
DENV-2_10F	Forward	GAGTAGGAACRARRCATGCA	3554-3573
DENV-2_10R	Reverse	TGTTGTGCAACTCACTTYCCATG	3968-3990
DENV-2_11F	Forward	GAAGTATCAATTAGCAGTGACYATCAT	3900-3926
DENV-2_11R	Reverse	AGTCCTGTTCTAATGAGTATRGTCA	4415-4439
DENV-2_12F	Forward	TCCAATTCTGTCAATAACAATATCRGA	4344-4370
DENV-2_12R	Reverse	CAGCCTCCTCCATAYGATATTA	4748-4769
DENV-2_13F	Forward	GGAACATTTCACACAATGTGGC	4651-4672
DENV-2_13R	Reverse	TCGTTTTATGGCTTCTCTRACTA	5141-5163
DENV-2_14F	Forward	CCAGACTGAAAAAAGCATYGAAGA	5019-5042
DENV-2_14R	Reverse	CATTGCTCTGAGGAAATGGRTC	5488-5509

DENV-2_15F	Forward	AGCTGGGATTTTYATGACRGC	5448-5468
DENV-2_15R	Reverse	CTTCTTTGTGCTGCACTRGAGT	5873-5894
DENV-2_16F	Forward	CATGAAACCAGTYATAYTRACAGAY	5805-5829
DENV-2_16R	Reverse	CTTTCTCCCTCTTTTGTCCARAT	6262-6284
DENV-2_17F	Forward	TGAAGGTATCAACTAYGCAGAC	6177-6198
DENV-2_17R	Reverse	CAGTTCACTAAGAGCATGRTTGTA	6496-6519
DENV-2_18F	Forward	CTTCCAACATTCATGACYCAGAA	6412-6434
DENV-2_18R	Reverse	TGATGCAGGACGTAGATCTATRT	6911-6933
DENV-2_19F	Forward	TATGTTGTCATAGCCATCCTYAC	6781-6803
DENV-2_19R	Reverse	TACAGTTGGGTTTTTCATRATGC	7244-7266
DENV-2_20F	Forward	CGTCCTYTTATTGGTAGCACAYTATG	7155-7180
DENV-2_20R	Reverse	GTCTCTCCTGTRTTRCCAGTTC	7571-7592
DENV-2_21F	Forward	GCTGGACTTCTCTTTTCYATYATGA	7522-7546
DENV-2_21R	Reverse	TARATTCCGMCCATATGTTGACAT	7915-7938
DENV-2_22F	Forward	GTTAAAGGCYTAACAAAAGGAGGR	7867-7890
DENV-2_22R	Reverse	CGTTTGGTGTCTCACTTTCAATYC	8366-8389
DENV-2_23F	Forward	AACATAGTGTCATCAGTGAAYATGA	8248-8272
DENV-2_23R	Reverse	TACCTGCTTTGTGAATTCTTCYCTR	8754-8778
DENV-2_24F	Forward	GATTGAGCAGACACCYAGRATGT	8727-8749
DENV-2_24R	Reverse	ATCCTCTATTGTGATTCTTGTGTC	9184-9207
DENV-2_25F	Forward	CAGCAGAGCTATATGGTACATGT	8979-9001
DENV-2_25R	Reverse	CAGTTTTKCACTGCWAYTTCTTC	9484-9506
DENV-2_26F	Forward	CAAAGAGGAAGTGGACARGTYG	9361-9382
DENV-2_26R	Reverse	TAAGCTCCACATTTGGGCGTA	9841-9861
DENV-2_27F	Forward	CATGTAGAAACCAAGATGAAYTGA	9749-9772
DENV-2_27R	Reverse	TGTWGTCTGTGTATTCYTCAYTGC	10193-10216
DENV-2_28F	Forward	TCATGGATTGGGCTAACAAGYAG	10114-10136
DENV-2_28R	Reverse	TAGTCCACTAAGCCATGCGT	10479-10498
DENV-2_29F	Forward	AGGTCGGATTAAGCCATAGTACG	10307-10329
DENV-2_29R	Reverse	TTGATTCAACAGCACCATTCCAT	10693-10715
DENV-3_1F	Forward	TTAGTCTACGTGGACCGACA	6-25
DENV-3_1R	Reverse	GCTGGCAATATCATCATGAGAC	405-426
DENV-3_2F	Forward	CAATATGCTGAAACGCGTGAGA	133-154
DENV-3_2R	Reverse	ATGTAAGGTTGYACCAGCAGT	628-648
DENV-3_3F	Forward	GATCTGGGAGARATGTGTGATG	554-575
DENV-3_3R	Reverse	TCGGTCTTCTGAAGCTCTATRT	1059-1080
DENV-3_4F	Forward	GTAGGAAACAGAGATTTYGTGGA	950-972
DENV-3_4R	Reverse	CTTCATTGTTAGTAAGATCATTTCATTG	1507-1534
DENV-3_5F	Forward	AAGTAGTGCAATATGAGAACCTY	1317-1339
DENV-3_5R	Reverse	GCACATTGCATAGCTCATCCC	1814-1834
DENV-3_6F	Forward	CACTTAAAGTGTAGACTYAAGATGGA	1772-1797
DENV-3_6R	Reverse	CTTCCGAATATTTGGTGCWCCAT	2231-2253
DENV-3_7F	Forward	ATTGGTATAAGAARGGRAGCTC	2097-2118
DENV-3_7R	Reverse	TCAGTTCATTGGCTATYTGCTTCC	2616-2639

DENV-3_8F	Forward	TGTGTGGAATTAGGTCRACRAC	2574-2595
DENV-3_8R	Reverse	CATGTGCAGGTTTTTACYTCTATGA	3063-3087
DENV-3_9F	Forward	CTGACATGGGCTAYTGGATAGA	3000-3021
DENV-3_9R	Reverse	TTTACACTCCCYGCTGAGACT	3460-3480
DENV-3_10F	Forward	ATATATGAATGGTGTTGCCGCT	3335-3356
DENV-3_10R	Reverse	ATTCCATTCGCCATTTGTTCAATG	3826-3849
DENV-3_11F	Forward	AGAAAATTTRCTGTTRGGAGTTG	3766-3788
DENV-3_11R	Reverse	CCAGTTATGACATAGCACGCTAT	4238-4260
DENV-3_12F	Forward	GGCTAGTTCYCTYCTTAGRAATGA	4174-4197
DENV-3_12R	Reverse	TCCTCCTCTTGTRACGTGC	4663-4681
DENV-3_13F	Forward	GAATTCCTGGRAAAACCCAAGTR	4599-4621
DENV-3_13R	Reverse	GACCCACGATGAAGRTCCATTA	5085-5106
DENV-3_14F	Forward	GGAGTTGGAAGAAGARATGTTYA	5044-5066
DENV-3_14R	Reverse	GGTAATCCAATCATTGCCYGAAT	5550-5572
DENV-3_15F	Forward	TCAGACTAACGCTCCAATYCA	5494-5514
DENV-3_15R	Reverse	CAGTGAGCATGGTCTTCRTCA	5959-5979
DENV-3_16F	Forward	GCAAGAAAATGACCAGTACATATTCA	5914-5939
DENV-3_16R	Reverse	AGGCACTCTTCCTATTTCTGTCA	6390-6412
DENV-3_17F	Forward	TTGATGCCCGCACYTATTCAGA	6303-6324
DENV-3_17R	Reverse	TAAGTATGCCTATYACGACATATG	6774-6797
DENV-3_18F	Forward	CATAATCCTGGAGTTTTTYATGATGGT	6694-6720
DENV-3_18R	Reverse	GTGAGAGTCAGAGGGTTCACT	7123-7143
DENV-3_19F	Forward	GTTGAGACAAAGGATGGCCRATA	7050-7072
DENV-3_19R	Reverse	GCTCCTTCTAAATAGCTCCCTCTA	7495-7518
DENV-3_20F	Forward	CCACAGGACCAATAACAACACT	7410-7431
DENV-3_20R	Reverse	CCTTTTGTGWATCCTCGYACT	7861-7881
DENV-3_21F	Forward	GGAAGAGGAGGCTGGTCATATTA	7811-7833
DENV-3_21R	Reverse	ACTCCTAAATCCACATCTTTCTCWATG	8314-8340
DENV-3_22F	Forward	ACTGTACAGRTTCACRATGACA	8278-8299
DENV-3_22R	Reverse	TTGCTGCGTTRGTTCTRACYT	8766-8786
DENV-3_23F	Forward	AGGGACAAGAARGGTYATGGR	8665-8685
DENV-3_23R	Reverse	TGAGAGTTTTCACGCGAGAAC	9052-9072
DENV-3_24F	Forward	TCGCTAGRGCYATATGGTAYATG	8970-8992
DENV-3_24R	Reverse	TTCTCTAGCRGATGRGGGTTYT	9465-9486
DENV-3_25F	Forward	TCACCAAYATGGAAGCCCAG	9396-9415
DENV-3_25R	Reverse	ACCCAATGGACTGGTACTGC	9908-9927
DENV-3_26F	Forward	CGCAAATGTGGAGTCTCATGTA	9840-9861
DENV-3_26R	Reverse	ACTTCTTCCGTACTGTGGCTTR	10307-10328
DENV-3_27F	Forward	CGAGGGAGCCATYTGGTAAAY	10249-10269
DENV-3_27R	Reverse	ACTTTGCAAGGAGGTACAGCTT	10545-10566
DENV-3_28F	Forward	TGACGTTAAAAGAAGAAGTCAGGC	10354-10377
DENV-3_28R	Reverse	CACCATTCCATTTTCTGGCGT	10667-10687
DENV-4_1F	Forward	GACCGACAAGGACAGTTCCAAA	18-39
DENV-4_1R	Reverse	CTTGTTGACAAGTGAAACGCCA	436-457

DENV-4_2F	Forward	CAACGATAACATTGYTGTGYTTGATT	400-425
DENV-4_2R	Reverse	CTACTCCTACGCATCGCATTC	937-957
DENV-4_3F	Forward	CCTACATGATTGGGCAAACAG	862-882
DENV-4_3R	Reverse	TTAACTTCAACCGATGGTGACCT	1437-1459
DENV-4_4F	Forward	CATGCAGTAGGAAATGACACATC	1383-1405
DENV-4_4R	Reverse	CTTCATTATTGACYTTYACCACTGT	1896-1920
DENV-4_5F	Forward	CTCAATTGAYAAAGARATGGCAGA	1856-1879
DENV-4_5R	Reverse	GTGATTCCTCCAACAGCTATG	2375-2395
DENV-4_6F	Forward	GTTCTTAGTGTTGTGGATTGGYA	2318-2340
DENV-4_6R	Reverse	GTCCGTCTATYAAAAATGTGYTRTT	2811-2835
DENV-4_7F	Forward	AGAAGACATGGGGRAARGCA	2767-2786
DENV-4_7R	Reverse	AATTTTCCAGATGCAGTGGTRGT	3321-3343
DENV-4_8F	Forward	CAGGAGGATTGTGACCAYAGAG	3285-3306
DENV-4_8R	Reverse	ATAAGATTAACCCTAATGATATTCCRTCA	3848-3876
DENV-4_9F	Forward	CTGATGGTAATAGGRATGGCCA	3783-3804
DENV-4_9R	Reverse	GGAGGGTAATCATGTTGGTTTCCT	4402-4425
DENV-4_10F	Forward	CTCAAGCCCAATCATAGAAGTGAA	4340-4363
DENV-4_10R	Reverse	CAATTTCTCCAGTRAGGGTYTTGA	4870-4893
DENV-4_11F	Forward	GAAAAAATCCRAAACAYGTCCAA	4832-4853
DENV-4_11R	Reverse	TATAAAGGTTGCATGRCACATGA	5299-5321
DENV-4_12F	Forward	GCTGTGAAATCAGARCACACAG	5259-5280
DENV-4_12R	Reverse	CTAAAGTTRGCCCCCATTTCAGATA	5752-5776
DENV-4_13F	Forward	CGAAAACGAAACTCACGGACT	5707-5727
DENV-4_13R	Reverse	TCTCTCCCTCTCTAGTCCARATT	6263-6285
DENV-4_14F	Forward	AATGGTGCTTCACAGGGGAAA	6205-6225
DENV-4_14R	Reverse	ATATGATTGAGGCCGYTATCCA	6687-6708
DENV-4_15F	Forward	TAGTAGCTTGCTCTGGGTRG	6650-6669
DENV-4_15R	Reverse	TCGTTGGGTTCACTTGRGAAT	7114-7134
DENV-4_16F	Forward	CTAATGGGGCTTGGAAAAGGAT	7041-7062
DENV-4_16R	Reverse	TGGATACAGCTATGGTYGTGTT	7458-7479
DENV-4_17F	Forward	ATTGACTTTGGCCACAGGAC	7397-7416
DENV-4_17R	Reverse	TTGCTCAGTGGGTTTGTAGAACA	7957-7979
DENV-4_18F	Forward	GCTGGAACTTGGTCAAACTYC	7921-7941
DENV-4_18R	Reverse	GACGCTTCTTCATAGCTYCCAT	8479-8500
DENV-4_19F	Forward	TTGGCACTATGATCATGAAAAYCC	8435-8458
DENV-4_19R	Reverse	CTTCCACTCCACTCCATGARTT	9066-9087
DENV-4_20F	Forward	CGCGGTTTCTGGAATTTGAAGC	9004-9025
DENV-4_20R	Reverse	CCATTTCTCAACCCTTTCTTTCA	9481-9503
DENV-4_21F	Forward	ATCCGCCAAATGGAAGCTGAA	9420-9440
DENV-4_21R	Reverse	CATGTTGTTCTGCTTGTTGGAAAC	9926-9949
DENV-4_22F	Forward	CCATGGCTATATGCTCAGCAG	9892-9912
DENV-4_22R	Reverse	AATATGCCACGCGTACAGCTT	10395-10415
DENV-4_23F	Forward	GCTCCTTTCGAGAGTGAAGGA	10236-10256
DENV-4_23R	Reverse	CCTGGATTCAACAACACCAATC	10622-10643

a For one sample, 2 tubes of PCR amplification with primer pool-1 and pool-2 separately were required. Odd-numbered and even-numbered primers were divided into primer pool-1 and pool-2, respectively.

b The genome position of DENV-1, 2, 3 and 4 were based on NCBI reference sequence NC_001477, NC_001474, NC_001475 and NC_002640, respectively.

TABLE S2 The GenBank Accession No. of the consensuses generated by NGS method and Sanger Sequencing method of the 31 DENV strains in this study.

Sample No.	Serotype	Genotype	Original	Consensus generated by NGS method with the new panel in this study		Consensus generated by Sanger Sequencing method		Consistency with Sanger Results (Bases with conflict)
				Length (bp) [a]	GenBank Accession No.	Length (bp)	GenBank Accession No.	
1	DENV-1	Genotype I	Cambodia	10481	ON908218	10648	ON867003	100.00% (0)
2	DENV-1	Genotype I	Cambodia	10449	ON911333	10426	ON907577	100.00% (0)
3	DENV-1	Genotype I	China	10493	ON908212	10648	MN923103	100.00% (0)
4	DENV-1	Genotype I	China	10493	ON908213	10175	ON873885	100.00% (0)
5	DENV-1	Genotype I	Myanmar	10582	ON908214	10624	ON881145	100.00% (0)
6	DENV-1	Genotype IV	Philippines	10601	ON908215	10660	ON907578	100.00% (0)
7	DENV-1	Genotype IV	Philippines	10486	ON908216	10645	MN923105	100.00% (0)
8	DENV-1	Genotype V	India	10578	ON908220	10638	MN923086	100.00% (0)
9	DENV-1	Genotype V	Pakistan	10570	ON908217	10477	ON873938	100.00% (0)
10	DENV-2	Asian-1	Cambodia	10528	ON908224	10572	ON887284	100.00% (0)
11	DENV-2	Asian-1	Cambodia	10586	ON908230	10573	ON890479	100.00% (0)
12	DENV-2	Cosmopolitan	Cambodia	10588	ON908228	10558	ON907579	100.00% (0)
13	DENV-2	Cosmopolitan	China	10672	ON908244	10574	MN923108	100.00% (0)
14	DENV-2	Cosmopolitan	China	10588	ON908226	10558	ON887640	100.00% (0)
15	DENV-2	Cosmopolitan	China	10671	ON908227	10604	ON888666	100.00% (0)
16	DENV-2	Cosmopolitan	China	10563	ON908229	10565	ON890422	100.00% (0)
17	DENV-2	Cosmopolitan	Fuji	10635	ON908222	10564	ON875316	99.99% (1)
18	DENV-2	Cosmopolitan	Niger	10542	ON908223	10570	ON907580	100.00% (0)
19	DENV-2	Cosmopolitan	Philippines	10478	ON908221	10582	ON907581	100.00% (0)
20	DENV-3	Genotype I	Bangladesh	10641	ON908231	10656	MN922033	100.00% (0)
21	DENV-3	Genotype I	Bangladesh	10641	ON908235	10606	ON907583	100.00% (0)
22	DENV-3	Genotype I	China	10642	ON908245	10638	ON900159	100.00% (0)
23	DENV-3	Genotype I	China	10637	ON908237	10656	MN922041	100.00% (0)
24	DENV-3	Genotype I	Thailand	10641	ON908238	10650	MK894338	100.00% (0)
25	DENV-3	Genotype III	Ethiopia	10608	ON908232	10656	ON890788	100.00% (0)
26	DENV-3	Genotype III	Maldives	10645	ON908234	10640	ON907582	100.00% (0)
27	DENV-3	Genotype III	Maldives	10529	ON908233	10648	ON890789	100.00% (0)
28	DENV-4	Genotype I	Myanmar	10370	ON908246	10386	ON891146	100.00% (0)
29	DENV-4	Genotype I	Cambodia	10584	ON908241	10324	ON891141	100.00% (0)
30	DENV-4	Genotype I	Cambodia	10583	ON908242	10322	ON891143	100.00% (0)
31	DENV-4	Genotype II	Malaysia	10600	ON908239	10295	ON907584	100.00% (0)

a The consensuses were generated on a coverage depth level of 20X or more with a primer-cutting process to match the design-length.

Table S3. DENV references used for the designing of primer panels.

No.	Collection date	Region	Length	Serotype	GenBank Accession No.
1	2013	Angola	10602	DENV-1	KF184975
2	2019	Benin	10646	DENV-1	MN600714
3	2019	Cambodia	10648	DENV-1	MN923099
4	2002	Chile	10735	DENV-1	EU863650
5	2016	China	10735	DENV-1	MG840574
6	2017	China	10735	DENV-1	MF683117
7	2019	China	10687	DENV-1	MN444624
8	2019	China	10646	DENV-1	MN923104
9	2019	China	10637	DENV-1	MN923082
10	2019	China	10646	DENV-1	MN923090
11	2019	China	10646	DENV-1	MN923083
12	2019	China	10645	DENV-1	MN923091
13	2019	Congo	10568	DENV-1	MN577472
14	2012	Gabon	10751	DENV-1	MG877557
15	2021	India	10706	DENV-1	OM666557
16	2019	Indonesia	10648	DENV-1	MN923100
17	2013	New Caledonia	10723	DENV-1	MN621140
18	2016	Philippines	10735	DENV-1	MG840567
19	2013	Singapore	10735	DENV-1	KJ806939
20	2015	Singapore	10714	DENV-1	MF033237
21	2016	Singapore	10714	DENV-1	MF033256
22	2016	Singapore	10714	DENV-1	MF033255
23	2020	Singapore	10637	DENV-1	MW295817
24	2019	Tanzania	10621	DENV-1	MN923102
25	2012	Thailand	10719	DENV-1	MG877554
26	2010	Venezuela	10711	DENV-1	MH450306
27	2017	Brazil	10646	DENV-2	MK517773
28	2016	Burkina Faso	10675	DENV-2	KY627762
29	2019	Burkina Faso	10665	DENV-2	MT982148
30	2019	Cambodia	10570	DENV-2	MN923114
31	2019	Cambodia	10573	DENV-2	OL414743
32	2019	Cambodia	10565	DENV-2	MN923122
33	2019	Cambodia	10697	DENV-2	OL412740
34	2013	China	10724	DENV-2	MF459663
35	2014	China	10724	DENV-2	MH827525
36	2014	China	10724	DENV-2	MH827531
37	2016	China	10723	DENV-2	MF043956
38	2017	China	10176	DENV-2	MH110572
39	2018	China	10558	DENV-2	MK564484
40	2019	China	10570	DENV-2	MN923120

41	2019	China	10574	DENV-2	MN923108
42	2019	China	10574	DENV-2	MN923116
43	2019	China	10567	DENV-2	MN923119
44	2019	China	10567	DENV-2	MN923121
45	2019	China	10568	DENV-2	MN923118
46	2014	Ecuador	10460	DENV-2	KY474334
47	1980	India	10591	DENV-2	JQ922553
48	2013	India	10625	DENV-2	MH822956
49	2014	Indonesia	10723	DENV-2	MH823208
50	2017	Maldives	10724	DENV-2	MH827551
51	1995	Peru	10713	DENV-2	EU056811
52	1997	Puerto Rico	10991	DENV-2	KF955373
53	2016	Seychelles	10724	DENV-2	MN272405
54	2011	Singapore	10723	DENV-2	MW512369
55	2016	Thailand	10670	DENV-2	LC410185
56	2019	Thailand	10551	DENV-2	MN923111
57	2019	Thailand	10711	DENV-2	MZ636804
58	2016	Vanuatu	10722	DENV-2	MH985859
59	2019	Vietnam	10574	DENV-2	MN923113
60	2017	Bangladesh	10666	DENV-3	LC436677
61	2017	Burkina Faso	10678	DENV-3	MT261978
62	2006	Cambodia	10525	DENV-3	HM181933
63	2008	Cambodia	10631	DENV-3	GU131946
64	2013	China	10707	DENV-3	MF682972
65	2017	China	10708	DENV-3	MN018388
66	2019	China	10642	DENV-3	MN227703
67	2019	China	10654	DENV-3	MN964274
68	2019	China	10654	DENV-3	MN922035
69	2015	Colombia	10707	DENV-3	MH544649
70	2016	Colombia	10706	DENV-3	MH544651
71	2009	India	10259	DENV-3	KU509281
72	2013	India	10579	DENV-3	MH822957
73	2016	India	10285	DENV-3	MG721064
74	2016	India	10619	DENV-3	MG721059
75	2016	Indonesia	10707	DENV-3	KY863456
76	2016	Indonesia	10707	DENV-3	MH823209
77	2013	Laos	10627	DENV-3	MF370226
78	2018	Malaysia	10642	DENV-3	MK894341
79	2018	Maldives	10650	DENV-3	MK894340
80	2017	Myanmar	10645	DENV-3	MW788885
81	2020	Myanmar	10656	DENV-3	MW295815
82	2008	Philippines	10312	DENV-3	KU509279
83	2019	Philippines	10654	DENV-3	MN922037
84	2019	Philippines	10654	DENV-3	MN922038

85	2015	Singapore	10675	DENV-3	KY921906
86	2015	Singapore	10667	DENV-3	KY921907
87	2018	Tanzania	10650	DENV-3	MK894339
88	1973	Thailand	10707	DENV-3	DQ863638
89	2011	Thailand	10261	DENV-3	KU509280
90	2014	Thailand	10681	DENV-3	KT424097
91	2010	Brazil	10604	DENV-4	JQ513332
92	2013	Brazil	10355	DENV-4	KP188565
93	2008	Cambodia	10656	DENV-4	JN638572
94	2018	Cambodia	10350	DENV-4	MK614090
95	2019	Cambodia	10337	DENV-4	MZ976858
96	2003	China	10617	DENV-4	KY670635
97	2018	China	10350	DENV-4	MK614093
98	2010	French Polynesia	10573	DENV-4	JQ915084
99	2014	Haiti	10649	DENV-4	KT276273
100	2009	India	10505	DENV-4	KU509287
101	2016	India	10652	DENV-4	MG272274
102	2014	Indonesia	10648	DENV-4	MH823210
103	2014	Malaysia	10331	DENV-4	MH051734
104	2020	Malaysia	10281	DENV-4	MW301595
105	2017	Myanmar	10547	DENV-4	MW788992
106	2017	Myanmar	10426	DENV-4	MW295825
107	2016	Papua New Guinea	10653	DENV-4	MH382789
108	2018	Philippines	10304	DENV-4	MK640208
109	2011	Singapore	10651	DENV-4	KP792537
110	2017	Thailand	10604	DENV-4	LC410203

TABLE S4 The DENV-specific primers and probes used for real-time PCR in this study.[a]

Name	Sequence (5'→3')	Note	Target
DENV-F	GCATATTGACGCTGGGAGAGA	forward primer	
DENV-R	GGCGTTCTGTGCCTGGAAT	reverse primer	DENV 1-4
DENV-P	CAGAGATCCTGCTGTCTC	probe	
DENV1-F	CAAAAGGAAGTCGTGCAATA	forward primer	
DENV1-R	CTGAGTGAATTCTCTCTACTGAACC	reverse primer	DENV-1
DENV1-P	CATGTGGTTGGGAGCACGC	probe	
DENV2-F	CAGGTTATGGCACTGTCACGAT	forward primer	
DENV2-R	CCATCTGCAGCAACACCATCTC	reverse primer	DENV-2
DENV2-P	CTCTCCGAGAACAGGCCTCGACTTCAA	probe	
DENV3-F	GGACTGGACACACGCACTCA	forward primer	
DENV3-R	CATGTCTCTACCTTCTCGACTTGTCT	reverse primer	DENV-3
DENV3-P	ACCTGGATGTCGGCTGAAGGAGCTTG	probe	
DENV4-F	TTGTCCTAATGATGCTGGTCG	forward primer	
DENV4-R	TCCACCTGAGACTCCTTCCA	reverse primer	DENV-4
DENV4-P	TTCCTACTCCTACGCATCGCATTCCG	probe	

a These primers and probes were recommended by the National 283 Health Commission of the People’s Republic of China (WS 216-2018, Diagnosis for dengue fever).
